# Environmental variables driving species and genus level changes in annual plankton biomass

**DOI:** 10.1093/plankt/fbz063

**Published:** 2019-12-19

**Authors:** Louise Forsblom, Jonna Engström-öst, Sirpa Lehtinen, Inga Lips, Andreas Lindén

**Affiliations:** 1 ENVIRONMENTAL AND MARINE BIOLOGY, ÅBO AKADEMI UNIVERSITY, Artillerigatan 6, 20520 ÅBO, Finland; 2 Marine Research Laboratory, MARINE RESEARCH CENTRE, FINNISH ENVIRONMENT INSTITUTE, Agnes Sjöbergin Latu 2, 00790 HELSINKI, Finland; 3 Bioeconomy team, NOVIA UNIVERSITY OF APPLIED SCIENCES, Raseborgsvägen 9, 10600 EKENäS, Finland; 4 DEPARTMENT OF MARINE SYSTEMS, TALLINN UNIVERSITY OF TECHNOLOGY, Akadeemia Rd. 15A, 12618 TALLINN, Estonia

**Keywords:** density dependence, population dynamics, phytoplankton, sampling bias, state-space models

## Abstract

Abiotic variables subject to global change are known to affect plankton biomasses, and these effects can be species-specific. Here, we investigate the environmental drivers of annual biomass using plankton data from the Gulf of Finland in the northern Baltic Sea, spanning years 1993–2016. We estimated annual biomass time-series of 31 nanoplankton and microplankton species and genera from day-level data, accounting for the average phenology and wind. We found wind effects on day-level biomass in 16 taxa. We subsequently used state-space models to connect the annual biomass changes with potential environmental drivers (temperature, salinity, stratification, ice cover and inorganic nutrients), simultaneously accounting for temporal trends. We found clear environmental effects influencing the annual biomasses of *Dinobryon faculiferum*, *Eutreptiella* spp., *Protoperidinium bipes*, *Pseudopedinella* spp., *Snowella* spp. and *Thalassiosira baltica* and indicative effects in 10 additional taxa. These effects mostly concerned temperature, salinity or stratification. Together, these 16 taxa represent two-thirds of the summer biomass in the sampled community. The inter-annual variability observed in salinity and temperature is relatively low compared to scenarios of predicted change in these variables. Therefore, the potential impacts of the presented effects on plankton biomasses are considerable.

## INTRODUCTION

In marine pelagic ecosystems, autotrophic nanoplankton and microplankton taxa are responsible for nearly all primary production. Many of these plankton species and genera have specific traits that can be of ecological, recreational or economic importance (McQuatters-Gollop *et al.,* 2017), e.g. activities such as fisheries and tourism can suffer from algal mass occurrences (Huisman *et al.,* 2018). Dominant species within the community can affect the whole ecosystem in many ways, by contributing to hypoxia, changing nutrient dynamics or driving food quality for consumers (Andersson *et al.,* 2015; McQuatters-Gollop *et al.,* 2017). When studying plankton dynamics using classes or other large taxonomic ranks, there is a risk that ecologically important information is overlooked by averaging out data, highlighting the need for species-specific analyses (Griffiths *et al.,* 2016; Hjerne *et al.,* 2019). This can occur if one taxon dominates the total biomass or if taxa with very different temporal patterns are combined (Rantajärvi *et al.,* 1998a; Hjerne *et al.,* 2019).

The impact of an environmental variable on population dynamics is typically largest when it is highly variable and affects population growth with a steep and monotonic functional response. The latter is most common in situations when populations are not experiencing optimum conditions (Eppley, 1972). Temperature, salinity, stratification and nutrients are key environmental variables for plankton population dynamics, and these variables are also influenced by anthropogenic pressures such as eutrophication and climate change (Suikkanen *et al.,* 2013; Andersson *et al.,* 2015). Annual mean temperatures in temperate regions are generally a few degrees below the predicted evolutionary stable conditions for the local phytoplankton taxa (Thomas *et al.,* 2012). Salinity stress can vary seasonally and influences growth performance of phytoplankton (Kirst, 1990) and determines the species composition (e.g. brackish-water environments can support taxa of both marine and freshwater origin). Stratification and ice conditions can have complex effects on plankton by indirectly influencing the onset of the spring bloom and thus interactions between species (Griffiths *et al.,* 2016; Hjerne *et al.,* 2019).

Our study area is located in the Gulf of Finland and a previous study in the area showed increasing class-level trends in biomasses of Cyanophyceae (cyanobacteria), Chrysophyceae and Prymnesiophyceae and a decrease in Cryptophyceae (Suikkanen *et al.,* 2013). All mentioned trends are suggested to be at least partially linked to the temperature increase in the area. Species-level studies investigating environmental effects in the area are available at least for the mixotrophic ciliate *Mesodinium rubrum* (Lips and Lips, 2017), the cyanobacteria *Aphanizomenon flos-aquae* (syn. A. flosaquae) and *Nodularia spumigena* (Kanoshina *et al.,* 2003), as well as the dinoflagellates *Peridiniella catenata* and the *Biecheleria baltica* complex (Klais *et al.,* 2013). One method for identifying such environmental drivers of biomass is including them as lagged candidate covariates into population models that alone can describe simple trends and autocorrelated temporal structure. This approach to time-series analysis is referred to as Granger causality in other contexts (Granger, 1969).

Many plankton studies use multivariate autoregressive models to identify community interactions along with abiotic drivers (Ives *et al.,* 2003; Hampton and Schindler, 2006; Scheef *et al.,* 2013; Griffiths *et al.,* 2016; Barraquand *et al.,* 2018). Data from marine systems, however, may show considerable observation error compared to freshwater systems (Hampton *et al.,* 2013; Scheef *et al.,* 2013), and autoregressive models can be extended to account for observation error by using a state-space model (SSM) approach. SSMs consider both stochastic population dynamics and sampling noise in the same model (Durbin and Koopman*,* 2012). Some plankton species display repeated patterns of occurrence annually, but local disturbances can cause high variability (Winder and Cloern, 2010; Scharfe and Wiltshire, 2019), which in combination with variable timing of sampling can complicate the estimation of reasonable annual abundances. Wind conditions can, for instance, vary substantially on short time scale and affect the vertical and horizontal distribution of species (Kanoshina *et al.,* 2003).

In this study, we go beyond trends, aiming to explicitly link changes in annual biomasses of 31 species and genera of auto-, mixo- and heterotrophic nano- and microplankton with environmental variables. We particularly study the effects of temperature, salinity, stratification, ice cover and nutrients. We apply SSMs for time-series analysis, accommodating observation errors, and estimating the environmental effects without relying on matching trends and autocorrelated temporal patterns, in line with the principle of Granger causality. Temperature is expected to influence most of the taxa in a positive manner, except for Cryptophyceae that are suggested to prefer cooler waters in the study area (Suikkanen *et al.,* 2013). We expect that salinity effects will be detected in taxa with freshwater or marine origin and that the effects will be negative and positive, respectively. We include both nutrients and stratification in the models with no *a priori* expectations. Additionally, we investigate if wind during the sampling day influenced the sampled biomass, simultaneously correcting for possible biases in the annual biomass estimates caused by wind or sampling date.

## METHOD

### Study area

**Fig. 1:**
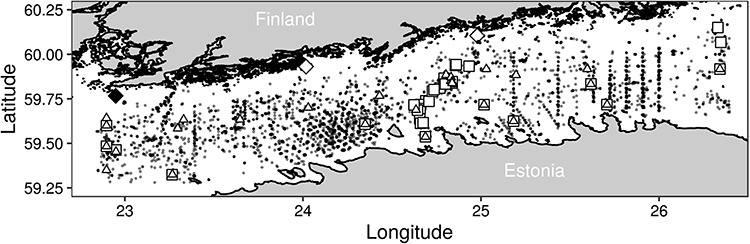
Plankton sampling locations used in the analysis (white square), locations of the wind stations (Bågaskär and Harmaja white diamond from left to right), location of the ice station (Russarö Island black diamond), nutrient sampling sites (white triangle) and the locations for temperature and salinity measurements (gray circles).

Our study area is the Gulf of Finland, a basin in the north-eastern part of the brackish-water Baltic Sea (Fig. 1). Salinity in the Baltic Sea decreases gradually towards the northern and eastern sub-basins, due to a very narrow connection to the Atlantic Ocean via the Danish straits. The plankton data were gathered from 31 locations in the Gulf of Finland, but some of them vary between years, as they have been sampled aboard commercial ships en route.

### Plankton data

The plankton data were compiled from different sources and have been collected as part of national phytoplankton monitoring schemes and Ferrybox/Algaline projects by the Estonian Marine Institute of University of Tartu, the Marine Systems Institute of Tallinn University of Technology and the Finnish Environment Institute (Table SI). Some of the data are available in the Gulf of Finland Year 2014 dataset and from the Finnish national database (http://www.syke.fi/avoindata). The data cover a period from 1993 to 2016 and consist of 1089 unique samples. The spatial and seasonal distribution of the sampling is shown in Fig. 1 and Fig. S1 respectively. Sampling has been carried out either by taking integrated samples from surface to 10 m (HELCOM, 2017), or by using the ferrybox systems (Rantajärvi and Leppänen, 1994; Jaanus *et al.,* 2009; Lips and Lips, 2017), installed on passenger ferries on the route between Helsinki and Tallinn. The ferrybox samples were collected at 4–5 m depth by an automated refrigerating sampler. All samples collected with both methods were analyzed using an inverted light microscope, according to the phytoplankton monitoring guidelines of HELCOM (2017), and the species nomenclature follows the Checklist of Baltic Sea Phytoplankton species (Hällfors, 2004). Biovolumes were calculated sample-wise based on size measurements and cell shape approximations by simple solids and converted to biomasses (wet weight μg L^−1^)(Olenina *et al.,* 2006). If there were counts for several size-classes within a taxon, biomasses of the size-classes were summed, resulting in one biomass per taxon and sampling event.

The taxa were selected for analysis based on their occurrence in the data. Selected taxa were required to have data from a minimum of 20 years, and we required a non-zero biomass three times annually. Further, the choice of taxa was done at the most specific taxonomic level accommodated by the data, assuming that the selected taxa were consistently identified by different microscopists during the study period. The used approach resulted in 24 autotrophic or mixotrophic phytoplankton taxa, as well as the mixotrophic ciliate *Mesodinium rubrum* and 6 heterotrophic plankton taxa (*Amphidinium crassum*, *Ebria tripartita*, *Katablepharis* spp., *Protoperidinium bipes*, *Protoperidinium brevipes, Telonema subtile*), fulfilling the given criteria. The selected taxa are presented in Table I together with their annual contribution to the total annual summer biomass.

**Table I:** Plankton taxa studied, their class, biomass contribution, sample size in the day-level observation model and the experienced physical conditions.

Class	Taxa	BM%	*n*	Med JD	temp	sal	strat
Chlorophyceae	*Monoraphidium contortum*	1.15	936	163	6.00 (0.21)	5.42 (0.06)	0.59 (0.04)
	*Oocystis* spp.	0.24	1089	238	16.24 (0.24)	5.53 (0.05)	1.42 (0.10)
	*Planctonema lauterbornii*	0.09	1067	280	16.04 (0.19)	5.47 (0.06)	0.96 (0.10)
Chrysophyceae	*Dinobryon faculiferum*	0.09	1067	157	5.50 (0.22)	5.45 (0.07)	0.55 (0.04)
	*Pseudopedinella* spp*.*	1.56	987	187	8.49 (0.18)	5.42 (0.06)	0.80 (0.04)
Ciliate	*Mesodinium rubrum*	11.05	1089	156	5.41 (0.21)	5.40 (0.06)	0.55 (0.04)
Cryptophyceae	*Hemiselmis* spp.	0.32	1086	233	16.12 (0.24)	5.52 (0.05)	1.44 (0.10)
	*Plagioselmis prolonga*	1.48	1083	228	15.86 (0.23)	5.50 (0.05)	1.42 (0.10)
	*Teleaulax* spp.	1.38	1083	239	16.29 (0.24)	5.53 (0.05)	1.41 (0.10)
Cyanophyceae	*Aphanizomenon* spp*.*	29.82	1089	197	10.33 (0.17)	5.45 (0.06)	0.99 (0.04)
	*Dolichospermum* spp*.*	3.69	1089	197	10.33 (0.17)	5.45 (0.06)	0.99 (0.04)
	*Nodularia* spp*.*	6.73	1089	208	13.23 (0.18)	5.52 (0.05)	1.25 (0.06)
	*Planktolyngbya* spp.	0.25	1078	260	16.67 (0.23)	5.57 (0.05)	1.25 (0.10)
	*Snowella* spp.	0.73	1089	252	16.64 (0.23)	5.59 (0.05)	1.30 (0.10)
	*Woronichinia* spp*.*	0.37	1089	284	15.63 (0.22)	5.47 (0.06)	0.88 (0.09)
Diatomophyceae	*Chaetoceros* spp.	2.18	1089	117	1.37 (0.15)	5.65 (0.07)	0.25 (0.02)
	*Skeletonema marinoi*	2.31	1089	114	1.27 (0.15)	5.66 (0.07)	0.26 (0.02)
	*Thalassiosira baltica*	3.23	1089	116	1.33 (0.15)	5.65 (0.07)	0.25 (0.02)
Dinophyceae	*Amphidinium crassum*	0.28	1089	188	8.61 (0.18)	5.43 (0.06)	0.81 (0.04)
	*Dinophysis acuminata*	3.86	1089	198	10.65 (0.17)	5.44 (0.06)	1.01 (0.04)
	*Heterocapsa rotundata*	0.78	1089	239	16.29 (0.24)	5.53 (0.05)	1.41 (0.10)
	*Heterocapsa triquetra*	7.21	1067	211	13.77 (0.19)	5.54 (0.05)	1.29 (0.07)
	*Peridiniella catenata*	11.52	1089	123	1.53 (0.16)	5.62 (0.07)	0.26 (0.02)
	*Protoperidinium bipes*	0.46	1089	126	1.70 (0.17)	5.61 (0.07)	0.26 (0.02)
	*Protoperidinium brevipes*	0.67	1056	161	5.84 (0.22)	5.45 (0.07)	0.58 (0.04)
Ebriidea	*Ebria tripartita*	1.19	1038	203	11.84 (0.17)	5.47 (0.06)	1.14 (0.06)
Euglenophyceae	*Eutreptiella* spp*.*	1.26	1089	187	8.49 (0.18)	5.42 (0.06)	0.80 (0.04)
Incertae sedis	*Katablepharis* spp*.*	0.32	1089	148	4.43 (0.21)	5.44 (0.07)	0.46 (0.04)
	*Telonema subtile*	0.05	1089	197	10.33 (0.17)	5.45 (0.06)	0.99 (0.04)
Prasinophyceae	*Pyramimonas* spp.	2.61	1089	201	11.3 (0.18)	5.44 (0.06)	1.09 (0.05)
Prymnesiophyceae	*Chrysochromulina* spp.	3.96	1035	208	13.23 (0.18)	5.52 (0.05)	1.25 (0.06)

BM% refers to the average percentage of the total sampled plankton biomass, calculated using raw data from May through September 1993–2016. The Julian day for the median biomass occurrence is presented according to the intra-annual model (Med JD). The means (intercepts from the GAMM) of temperature (temp), salinity (sal) and stratification (strat) experienced by each taxa 0–59 days prior to Med JD, together with their SE in parenthesis Dolichospermum spp. includes pelagic taxa which were earlier included into Anabaena spp. Skeletonema marinoi includes diatoms which were earlier identified as S. costatum in the Baltic Sea.

### Environmental covariate data

Most of the environmental covariates used in the analysis were gathered by the Finnish Environment Institute (http://www.syke.fi/avoindata), the Finnish Meteorological Institute (FMI) and the Estonian Marine Institute. Data on salinity and temperature (°C) were downloaded from the data portal of the International Council for the Exploration of the Sea data portal (ICES, 2014). These data were from a larger area compared to the plankton data (Fig. 1). For each sample set of temperature and salinity, the difference in stratification between the surface and 20 m (*E*) was derived from salinity, temperature and depth data and was calculated similarly to Kuosa *et al.* (2017) (Equation (1)).

(1) $$ \mathrm{E}={\sigma}_{T\left(20\mathrm{m}\right)}-{\sigma}_{T\left(\mathrm{surface}\right)} $$

where *σ_T_* is water density anomaly in kg m^−3^ calculated from measurements at ~20 m and at the surface of the respective sample. A larger difference between layers indicates more stratified conditions. The temperature, salinity and stratification data were subsequently used to construct annual taxon-specific anomalies using observations from a 60 day time-window (0–59 days) before the median day of occurrence of the focal taxon (Table I). A detailed description on the construction of these anomalies, as well as the number of data points used annually for each taxon, is available in the Supplementary material (Figs S3–S5). The day when a species’ seasonal biomass reaches its median, i.e. when the fitted daily cumulative biomass reaches 50%, was derived from the observation model and is presented in Table I.

Nutrient concentration data (μM) were averaged from 0 to 10 m and originated from multiple locations (Fig. 1). To achieve a complete time series, one additional station in the Baltic Proper was used in 1996. DIN (nitrate NO_3_^−^, nitrite NO_2_^−^ and ammonium NH_4_^+^), DIP (phosphate PO_4_^3−^) and silicate (SiO_4_) concentrations vary, but they are usually below detection level during summer (Suikkanen *et al.,* 2007); therefore, measurements obtained during the preceding winter, between November and March, were used to calculate annual averages of DIN, DIP and silicate (Fig. S2). There were 278 nutrient samples for each nutrient, with 1–16 samples annually (mean 7.3, standard deviation [SD] 4.1). Data on the number of days with more than 10% ice cover (Kalliosaari, 1978, 1982; Kalliosaari and Seinä, 1987; Seinä and Kalliosaari, 1991; Seinä *et al.,* 1996, 2001, 2006; J. Vainio, unpublished data) were collected at the weather station on Russarö Island, Hanko, Finland. Mean daily wind velocity data (m s^−1^) were collected at the weather station on Bågaskär, Ingå, Finland, and for the 4 days when data were missing from this site, additional measurements were used from Harmaja, Helsinki, Finland (Fig. 1).

All annual covariates were z-scored prior to the analysis, i.e. the sample mean was subtracted from all values, and the result was divided by the sample SD. This facilitates direct comparison of their relative effects on the dynamics of inter-annual relative biomass, as well as comparison between taxa (Schielzeth, 2010). The unit of a z-scored variable is SD, regardless of its original unit. The wind variable was likewise z-scored to facilitate comparison between taxa. The original means for temperature, salinity and stratification are available in Table I, and selected means and SD are available in Table II, while the rest are all available in the supplementary material (Table II).

**Table II:** Specifications and statistics of the most parsimonious models with environmental covariates included

Taxon	Env. cov.	*β*_1_ (trend)	*β_2_* (env. cov.)	Cov. $\overline{x}$ (SD)	***φ***	${\sigma}_{\mathrm{Proc}}^2$	ln*L*	*n*	ΔAICc
*Aphanizomenon* spp.	temp	0.15 (0.11)	0.20 (0.10)	10.33 (0.75)	**−0.10 (0.23)**	0.11 (0.04)	−12.59	24	0.86
*Chaetoceros* spp.	DIP	−0.05 (0.13)	**−0.27 (0.11)**	0.74 (0.16)	**−0.09 (0.29)**	0.15 (0.07)	−22.47	24	1.84
*Chaetoceros* spp.	strat	−0.14 (0.13)	**0.23 (0.12)**	0.25 (0.08)	**−0.23 (0.29)**	0.16 (0.08)	−22.90	24	0.98
*D. faculiferum*	sal	−0.15 (0.20)	**0.49 (0.20)**	5.45 (0.28)	**0.48 (0.23)**	0.51 (0.23)	−30.92	23	3.08
*D. faculiferum*	temp	0.17 (0.26)	**−0.60 (0.24)**	5.50 (1.03)	**0.53 (0.23)**	0.54 (0.24)	−31.19	23	2.54
*D. acuminata*	temp	0.10 (0.09)	0.17 (0.09)	5.44 (0.76)	**0.08 (0.28)**	0.06 (0.03)	−9.09	24	0.03
*E. tripartita*	strat	0.02 (0.15)	**−0.32 (0.14)**	1.14 (0.25)	**0.56 (0.21)**	0.31 (0.11)	−24.40	24	1.93
*Eutreptiella* spp.	temp	**−0.23 (0.10)**	**0.69 (0.10)**	8.49 (0.85)	**−0.04 (0.14)**	0.07 (0.04)	−13.45	24	21.7
*Hemiselmis* spp.	strat	**−0.56 (0.25)**	**−0.33 (0.16)**	1.44 (0.48)	**0.60 (0.19)**	0.35 (0.13)	−25.64	24	1.39
*H. rotundata*	sal	−0.18 (0.15)	**0.29 (0.14)**	5.53 (0.22)	**−0.15 (0.24)**	0.30 (0.11)	−22.51	24	1.26
*H. triquetra*	temp	0.31 (0.32)	−0.59 (0.32)	13.77 (0.78)	**−0.16 (0.21)**	1.57 (0.47)	−39.49	23	0.49
*M. rubrum*	sal	**0.61 (0.28)**	−0.22 (0.13)	5.40 (0.27)	**0.45 (0.19)**	0.23 (0.11)	−25.68	24	0.02
*P. bipes*	sal	−0.08 (0.13)	**0.45 (0.12)**	5.61 (0.29)	**0.46 (0.22)**	0.12 (0.08)	−20.60	24	8.32
*Pseudopedinella* spp.	sal	0.10 (0.13)	**−0.32 (0.11)**	5.42 (0.25)	**0.67 (0.19)**	0.18 (0.09)	−18.26	24	4.95
*Snowella* spp.	strat	**−1.06 (0.44)**	**−0.58 (0.25)**	1.30 (0.45)	**0.37 (0.24)**	0.70 (0.30)	−35.22	24	2.59
*Snowella* spp.	temp	**−0.84 (0.45)**	**−0.54 (0.23)**	16.64 (1.03)	**0.38 (0.24)**	0.62 (0.29)	−35.23	24	2.57
*Teleaulax* spp.	sal	−0.03 (0.12)	0.19 (0.11)	5.53 (0.22)	**0.44 (0.21)**	0.19 (0.06)	−15.96	24	0.44
*Teleaulax* spp.	strat	−0.07 (0.11)	−0.19 (0.11)	1.41 (0.47)	**0.39 (0.22)**	0.19 (0.06)	−16.09	24	0.19
*T. baltica*	ice	−0.28 (0.15)	**−0.54 (0.14)**	35.80 (35.11)	**−0.14 (0.21)**	0.13 (0.09)	−22.03	24	9.61
*Woronichinia* spp.	strat	**−0.59 (0.36)**	**−0.63 (0.32)**	0.88 (0.39)	**0.38 (0.24)**	1.24 (0.44)	−40.04	24	1.26
*Woronichinia* spp.	temp	**−1.02 (0.39)**	**0.68 (0.34)**	15.63 (0.95)	**0.27 (0.23)**	1.20 (0.43)	−40.08	24	1.19

The estimated parameters (and SE) are the partial trend (*β*_1_) and the covariate effect (*β*_2_), the autoregressive coefficient (*φ*) and process error variance (${\sigma}_{\mathrm{Proc}}^2$). Parameter estimates and their SE are bolded when the 95% CI do not cross zero (*β*_1_ and *β*_2_) or one for the autoregressive parameter (*φ*). The sample means and SDs used for z-scoring the environmental covariates are reported in the table (Cov $\overline{x}$ (SD)). For the continuous variable year (accounting for the partial trend), the sample means ($\overline{x}$) and SD depend on time-series length: for *n* = 23, $\overline{x}$= 12 and SD = 6.78; for *n* = 24, $\overline{x}$= 12.5 and SD = 7.07. Further presented statistics are the maximized log likelihood (ln*L*), the number of years (*n*) and the AICc difference of the null model compared to the focal model (ΔAICc).

### Strategy of analysis and model selection

We used a two-step approach, where we first fit a generalized additive mixed model (GAMM) for each taxon to day-specific biomass (further details in Day-level observation model), with the main goal to extract log-scale annual biomass estimates for each taxon, as well as their estimated uncertainties (standard errors [SE]). As biomass varies with sampling day (phenology) and may be affected by wind, we accounted for these in the GAMM. In a second step, we investigated the dynamics of annual relative log-scale biomass and, particularly, the effects of environmental covariates (further details in Inter-annual model for biomass dynamics). This was done using first-order autoregressive SSMs, with the estimated annual log-scale biomasses and SE as the input. According to Knape *et al.* (2013), state-space modeling, which uses time series of estimated annual abundances and their associated SE as input, typically provides reliable results. Although this approach ignores uncertainty in the estimated SE, simulations by Knape *et al.* (2013) indicated similar or even better results than in a case, where replicates were explicitly included in the observation model. Here, the two-step approach facilitates using a more complex and realistic observation model for estimating biomass.

The candidate SSMs were evaluated taxon-wise. They always included a uniform trend (study year as a covariate) and, to avoid overparameterization, at most one additional environmental covariate affecting biomass dynamics. The support for inclusion of a covariate in the model was evaluated using information theoretic model selection, applying Akaike information criterion for small sample size, AICc (Burnham and Anderson, 2002), using the number of years as sample size. Models were considered relevant if AICc was at least 2 units smaller, and indicative if AICc was smaller at all, compared to the null model with no environmental covariate. Whenever models including a covariate effect were more parsimonious compared to the null model, we further illustrated parameter uncertainty using unbiased 95% confidence intervals (CI) based on a parametric bootstrap with 1000 resampling events.

Effects of salinity, temperature or stratification were considered for all taxa using the taxon-specific anomaly time-series. These time-series were tailored to fit the time-window prior to the median biomass of each taxon. Additional covariates were considered on a taxon-specific basis, conditional on the timing of the median biomass of the taxa, which was calculated from the observation model (Table I). Candidate models for taxa that reach median biomass prior to 15 June (Julian day 166) included mean winter time measures of DIN and DIP and number of days with ice cover. Average wintertime silicate was only considered for diatoms and *Ebria tripartita*. All covariate combinations considered are listed in the supplementary material.

### Inter-annual model for biomass dynamics

The model for between-year biomass dynamics was fit using the MARSS package in R for fitting multivariate autoregressive SSMs (Holmes *et al.,* 2012, 2018). Each taxon was modeled separately, using the observation time series of relative annual biomasses centered to zero mean (*y* = (*y*_1_, *y*_2_, …, *y_t_*, …, *y_n_*); response variable in Equation (2)), which originates from the day-level observation model (GAMM). The observation time series, in turn, is assumed to arise from one underlying population state (*x* = (*x*_1_, *x*_2_, …, *x_t_*, …, *x_n_*)). The full model is:

(2, 3) $$ {y}_t={{x}}_t+{\nu}_t;\kern1em{\nu}_t \sim\!\! N\left(0,{\sigma}_{\rm{{Obs}}_t}^2\right) $$

(4, 5) $$ {x}_t={\varphi{x}}_{{t}\hbox{--} 1}+{{Z}\,}_t\beta +{\omega}_t;\kern1em{\omega}_t \sim\!\! N\left(0,{\sigma}_{{Proc}}^2\right) $$

(6) $$ {x}_1\sim\!\! N\left({y}_1,{\sigma}_{\rm{{Obs}}_1}^2\right) $$

According to Equations (2 and 3), the natural logarithm of the annual estimated relative biomass (*y_t_*) for time-step *t* is assumed to be measured with error but on average unbiased, being the true value *x_t_* plus normally distributed i.i.d. random noise *υ_t_* with time-varying observation error variance ${\sigma}_{\mathrm{Obs}_\mathrm{t}}^2$. The annual squared SE from the GAMM were used in the SSM as ${\sigma}_{\mathrm{Obs}_\mathrm{t}}^2$. The density-dependent process model (Equations (4–6)) contains an autoregressive coefficient (*φ*), the time series of z-scored covariates arranged in the columns of matrix Z (row *t* in the matrix is denoted Z*_t_*), their effects (*β*; column vector with the temporal trend effect *β*_1_, and optionally the environmental covariate effect *β*_2_) and the process error *ω_t_* with estimated variance ${\sigma}_{\mathrm{Proc}}^2$. The initial state *x*_1_ is assumed to be normally distributed with mean *y*_1_ and variance ${\sigma}_{\mathrm{Obs}_1}^2$, the latter being set to the squared SE of *y*_1_. The parameters estimated are *φ*, *β*_1_, *β*_2_ and ${\sigma}_{\mathrm{Proc}}^2$*.*

The SSM was fit using the expectation maximization algorithm (Holmes, 2013). To find good starting points for the searches, a Markov chain Monte Carlo routine with 100 starting conditions was implemented for each model. The SE for the SSM parameter estimates were calculated using parametric bootstrap with 1000 resampling events, however, only for the most parsimonious models that included relevant covariate effects. Otherwise, SE reported in the supplementary material were computed based on the Hessian of the negative log-likelihood at its minimum. The standardized residuals of the most parsimonious models—for both the observation- and process model part of the SSM—were visually investigated as autocorrelation function plots and quantile–quantile plots.

### Day-level observation model

The purpose of the intra-annual observation model was to estimate the annual log-scale mean biomasses and their sampling variances, to be subsequently used as input in the SSM (see *y* in Equation (2)). Simultaneously, we corrected for potential effects on daily biomass data induced by wind, sampling day and sampling method (integrated sampling or the ferrybox method). For this purpose we applied a GAMM, with the daily scale biomass observations as the response variable, applying a log link function and Tweedie error distribution with an estimated power parameter (Wood, 2011; Wood *et al.,* 2016). Patterns of phenology, common for all years, were modeled with a smoothing function (cyclic penalized cubic regression spline) of sampling day (Julian day). This means that we accounted for a repeated annual pattern or phenology, with the underlying simplification that the pattern is the same each year. Further adding the factor variable year to the model, gave us annual biomass estimates and associated SE. Daily z-scored wind velocity was included as a covariate. Sampling method and the interaction between sampling method and wind were included to distinguish between ferrybox and integrated sampling and possibly between method-specific effects of wind velocity. Finally, the factor variable location was included in the model with random effects on the intercepts. The models were fitted using the package mgcv in R (Wood, 2017b). Years with only zeroes were removed prior to analysis as the estimated value would go to minus infinity.

Due to mixing by the moving ship, the ferrybox samples are regarded to represent the upper ca. 10 m surface layer similarly as the integrated surface to 10 m samples (Rantajärvi *et al.,* 1998b; Kanoshina *et al.,* 2003). However, wind conditions along with the sampling method may cause different taxon-specific effects since the optimum depth into which biomass tends to aggregate varies between taxa and since taxa also have different modes of mobility (Hajdu *et al.,* 2007; Lips and Lips, 2014). Calm wind can for example lead to cyanobacterial accumulation at the sea surface, which in turn can lead to underestimation of *Nodularia* spp. biomass during intensive blooms (Kanoshina *et al.,* 2003; Lips and Lips, 2008). Since ferries are constantly in motion, ferrybox sampling is more exposed to turbulence compared to the integrated sampling. Thus, we expect that wind affects the biomass results from the ferrybox sampling to a lesser extent then when sampling has been done using the integrated sampling method.

The model fit was investigating graphically by looking at quantile–quantile graphs and histograms of the residuals, residuals plotted against the linear predictor and by refitting the residuals in an identity link Gaussian GAM against Julian day to look for unexplained patterns (Wood, 2017a). For this purpose, we used Dunn–Smyth residuals (Dunn and Smyth, 1996). We also assessed the assumption of constant phenology, by investigating how the most extreme positive outliers (>2) were distributed in time—more specifically we calculated the correlation between Julian day and year of those observations.

All data processing, analyses and visualization were done in R (R Core Team, 2017). In addition to MARSS and mgcv, the following packages were used: plyr (Wickham, 2016a), reshape2 (Wickham, 2016b), data.table (Dowle *et al.,* 2017), gamm4 (Wood and Scheipl, 2017), oce (Kelley *et al.,* 2017) and ggplot2 (Wickham and Chang, 2016).

## RESULTS

**Fig. 2:**
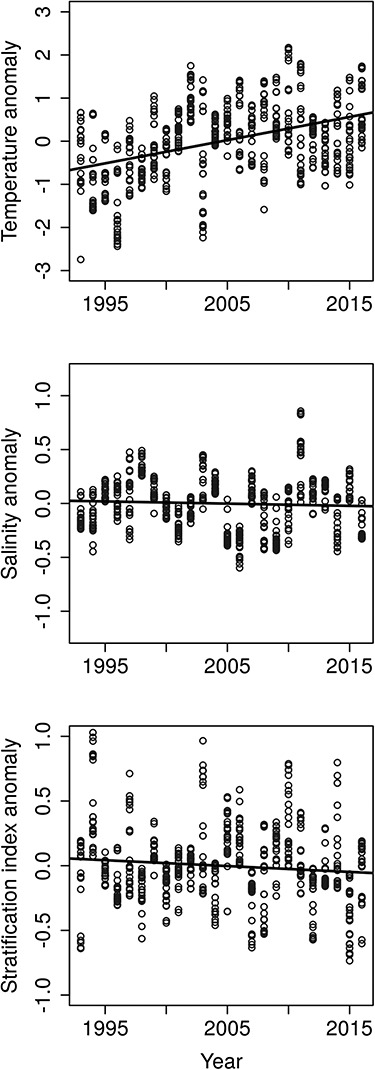
Annual modeled anomalies from the GAMMs for temperature, salinity and stratification index for all studied taxa. The line represents average annual anomalies regressed against year.

During the study period (years 1993–2016) temperature increased in the study area, while salinity and the stratification index slightly decreased (Fig. 2). The annual mean conditions experienced by the taxa 0–59 days before reaching median biomass, varied between 1.3 and 16.7°C, salinities between 5.4 and 5.7, and the stratification index between 0.3 and 1.4 units (Table I).

### Annual environmental drivers

**Fig. 3:**
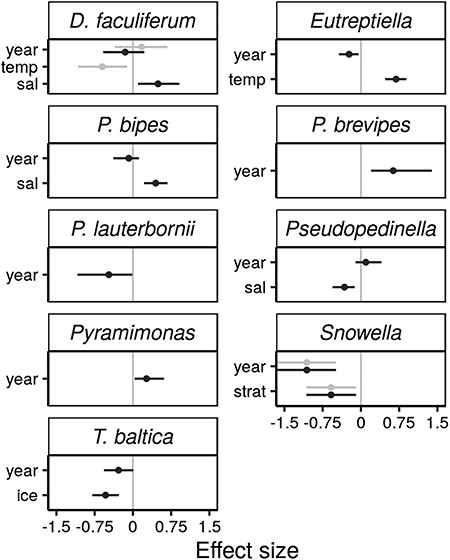
Estimated parameter effects of the z-scored environmental covariates on the annual relative biomasses in the most parsimonious models (black). Competing models are shown in gray. The standardized effects are comparable between covariates and taxa and directly proportional to their impact in practice on taxon-specific biomasses. The error bars indicate 95% CI. Covariates: temp = temperature, sal = salinity, strat = stratification index, DIN = dissolved inorganic nitrogen, DIP = dissolved inorganic phosphorous, ice = number of days with ice cover. All models also include a partial temporal trend (year).

The time-series of average annual log-scale biomasses had reasonably small SE in relation to the annual variation in biomass (Fig. S6). Using these time-series as response variables in SSMs, one taxon at a time, we detected environmental effects in 16 of 30 taxa. One of 31 initially considered species—*Peridiniella catenata*—was disregarded from the between-year analysis (SSM) due to convergence issues. For six taxa, results showed clear evidence for annual environmental effects (AICc at least 2 units smaller compared to the null model): *Dinobryon faculiferum*, *Eutreptiella* spp., *Protoperidinium bipes*, *Pseudopedinella* spp., *Snowella* spp. and *Thalassiosira baltica* (Fig. 3); whereas results showed weak evidence for ten taxa (AICc smaller than in the null model): *Aphanizomenon* spp., *Chaetoceros* spp., *Dinophysis acuminata, Ebria tripartita*, *Hemiselmis* spp., *Heterocapsa rotundata*, *Heterocapsa triquetra*, *Mesodinium rubrum*, *Teleaulax* spp. and *Woronichinia* spp. Five taxa had two models with environmental covariates that were more parsimonious than the null model: *D. faculiferum*, *Chaetoceros* spp., *Teleaulax* spp., *Snowella* spp. and *Woronichinia* spp. The 16 taxa together represent 65.5% of the summer biomass in the sampled community, *Aphanizomenon* spp. contributing the most (29.8%). The top ranked models and their parameter estimates are presented in Table II and the rest are included in Supplementary Table III.

Most effects were related to temperature, salinity or stratification. In general, we expected temperature effects on biomasses to be positive. However, temperature had effects (including tendencies) in both directions: four taxa with positive and three with negative effects. Temperature effects were not observed in any of the investigated cryptophytes (class Cryptophyceae). We expected salinity to have variable effects on biomass, and we found four positive effects as well as two negative effects. Interestingly, all taxa with positive effects of salinity had negative partial trends (not always significant though) indicating a temporal decrease in biomass, and vice versa (Fig. 2). Models including the stratification index had only one taxon with a positive effect and four taxa with negative effects on biomass. Nutrients and ice only affected one taxon each. The biomass of *Chaetoceros* spp. decreased with increasing DIP, and the biomass of *Thalassiosira baltica* decreased with an increasing number of ice days. For *Protoperidinium brevipes*, *Planctonema lauterbornii* and *Pyramimonas* spp., the results showed decreasing and increasing partial trends indicating changes in biomass, but environmental effects were not detected (Fig. 3). The remaining taxa had neither clear trends, nor environmental effects in their most parsimonious models (Table SIII). This was the case for *Dolichospermum* spp., *Nodularia* spp., *Planktolyngbya* spp., *Oocystis* spp., *Skeletonema marinoi*, *Amphidinium crassum*, *Plagioselmis prolonga*, *Chrysochromulina* spp., *Katablepharis* spp. and *Telonema subtile*. Considering only the most parsimonious models, all taxa showed density dependent dynamics (*φ* < 1) of variable strength, which implies a statistical return tendency at the annual level towards the average, differing from a pure random walk. The parameter estimates varied between −0.22 and 0.67 for the highest ranked models (Table II).

### Day-level observation model

The results presented above were based on the time series of average biomasses estimated using the day-level GAMM. The length of the time-series varied from 23 to 24 years. The results below reflect the daily effect of sampling on the observability of the taxon.

**Fig. 4:**
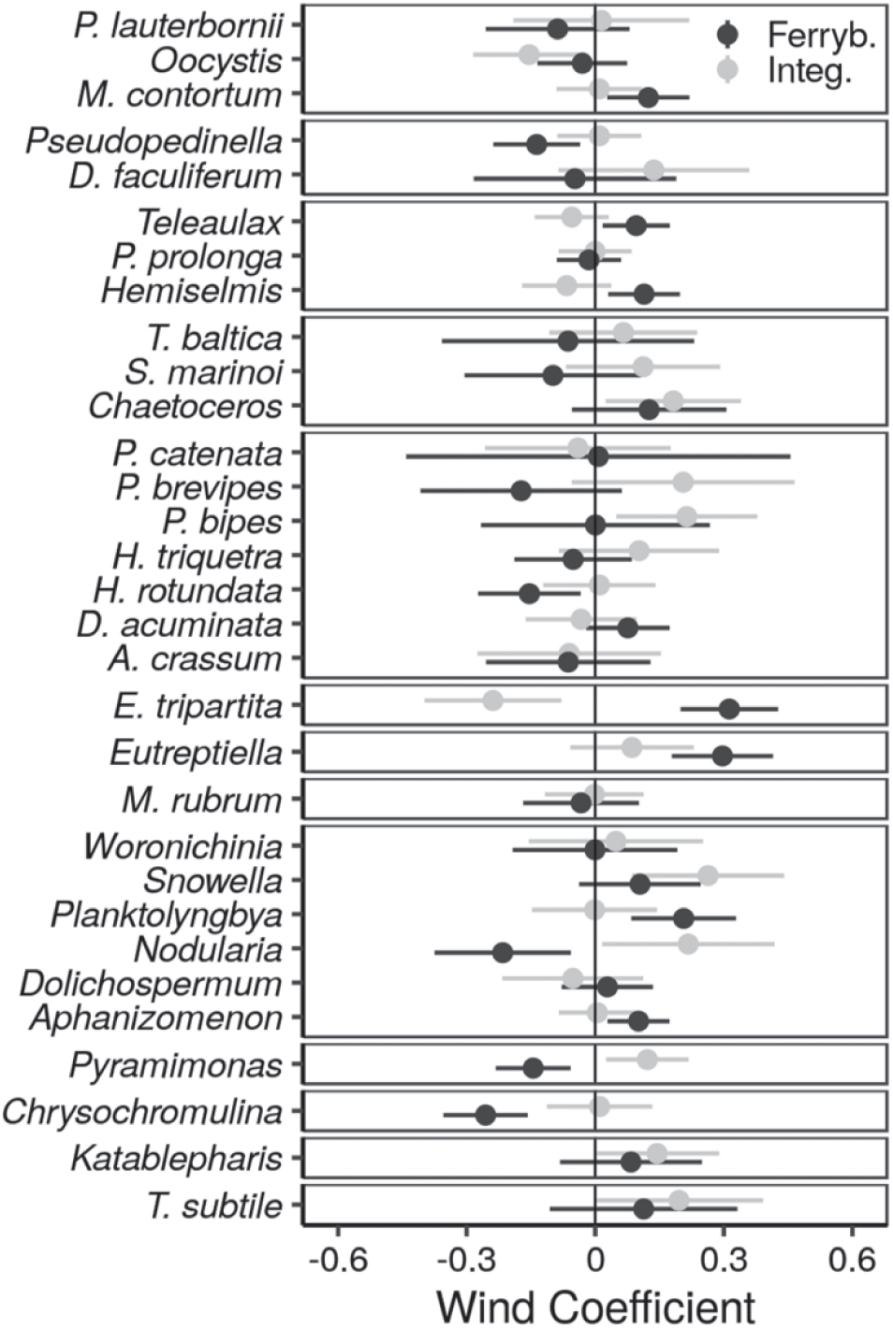
Estimated effects of the z-scored wind ($\overline{x}$ = 5.4 m s^−1^, SD = 2.1 m s^−1^) on the sampled log-transformed plankton biomasses (μg L^−1^) for integrated sampling (black) and ferrybox sampling (gray), based on the day-level GAMM analysis. The error bars are 95% CI (±1.96 × SE). The standardized effects are comparable between covariates and taxa, and directly proportional to their impact in practice on taxon-specific biomasses.

The wind during sampling had a significant effect (*P* < 0.05) on the observed biomass of 16 out of 31 taxa with either the integrated sampling method, the ferrybox sampling method, or both (Fig. 4). There were no consistent effects within classes, but the biomasses of *Pseudopedinella* spp., *H. rotundata*, *Nodularia* spp., *Chrysochromulina* spp. and *Pyramimonas* spp. decreased with increasing wind during ferrybox sampling, while the biomasses of *Aphanizomenon* spp., *E. tripartita*, *Eutreptiella* spp., *Hemiselmis* spp., *Monoraphidium contortum*, *Planktolyngbya* spp., and *Teleaulax* spp. were positively affected by wind. In samples taken using the integrated sampling method, the biomasses of *Nodularia* spp., *Chaetoceros* spp., *P. bipes*, *Snowella* spp., and *Pyramimonas* spp. were positively influenced by wind, whereas the biomasses of *E. tripartita* and *Oocystis* spp. decreased with increasing wind. For 10 taxa, wind effects were inconsistent between methods, i.e. there were significant (*P* < 0.05*)* interactions between wind and method. These were *Chrysochromulina* spp., *E. tripartita*, *Eutreptiella* spp., *Hemiselmis* spp., *Nodularia* spp., *P. brevipes*, *Planktolyngbya* spp., *Pseudopedinella* spp., *Pyramimonas* spp., and *Teleaulax* spp. For seven taxa, the biomass sampled using the integrated sampling method was consistently higher compared to the ferrybox method (*P* < 0.05). These taxa were *E. tripartita*, *Chaetoceros* spp., *M. contortum*, *D. faculiferum*, *Pseudopedinella* spp., *A. crassum*, and *T. subtile* (Table SIV).

The model fit investigation indicated a statistically significant (*P* < 0.05) negative correlation between Julian day and year of the most extreme positive outliers in eight of the investigated taxa: *Dolichospermum* spp., *E. tripartita*, *M. rubrum*, *Oocystis* spp., *Pseudopedinella* spp., *Skeletonema marinoi*, *Teleaulax* spp., and *T. subtile*, indicating an advanced (left-shifted) phenology over time. On the other hand, *Nodularia* spp. had significant positive correlation coefficients, indicating later phenology (Fig. S7). Among 16 taxa with environmental effects, four showed significant correlations: *E. tripartita*, *M. rubrum, Pseudopedinella* spp. and *Teleaulax* spp.

## DISCUSSION

### Annual environmental effects

We found environmental effects on the annual taxon-specific biomasses in half of the studied taxa, which together contribute to approximately two-thirds of the summer biomass. The effects were mostly linked to temperature, salinity and stratification (Fig. 3, Table II). Reliably linking covariates to the dynamics of natural populations is essential for understanding the drivers of abundance or biomass, even in cases where a species’ ecological requirements and tolerances are known from experimental studies. An environmental variable, which affects stress levels, survival or reproduction in artificial conditions, can in practice be irrelevant for population dynamics regionally if the variable shows minor fluctuations in nature or if it varies in a range within the optimal window for the species (see e.g. Jochem, 1990). Furthermore, some environmental variables may be irrelevant for large-scale annual population dynamics if the population response is temporally short or spatially restricted (Rantajärvi *et al.,* 1998a; Griffiths *et al.,* 2016).

Phytoplankton should be able to adapt to the local mean conditions as their maximum evolutionary stable temperatures in temperate regions are often above that of the mean of their local environment (Thomas *et al.,* 2012). Four of the selected taxa (*Aphanizomenon* spp., *D. acuminata, Woronichinia* spp. and *Eutreptiella* spp.) showed positive effects, indicating that the optimum temperature was not achieved. The negative effects detected, however, might suggest that the temperatures were above the optimum for *Heterocapsa triquetra*, *Snowella* spp. and *D. faculiferum.* If temperatures are above the optimal conditions, a rapid decline can be expected, compared to a less abrupt response below the optimum (Eppley, 1972). In a laboratory experiment, Yamaguchi *et al.* (1997) showed the optimum growth rate conditions for *H. triquetra* were 15°C. The salinity conditions in their study did not overlap with those experienced by *H. triquetra* in our study, but its salinity tolerance was noted to be broad. More interestingly, the optimum temperature range was quite narrow, spanning circa 5°C. In our study, the mean temperatures experienced by *H. triquetra* were ~14°C, which is close to the previously reported optimum. Another explanation for a negative effect of temperature on *H. triquetra* may be connected with the positive effect of temperature on stratification. *H. triquetra* biomass seems to be very much connected with the ability to perform vertical migrations in the water column and this ability is very much influenced by the stratification parameters and background hydrodynamical processes (Lips and Lips, 2014). We used *H. triquetra* as an example as it has been better studied compared to both *D. faculiferum* and *Snowella* spp., even if its temperature effect was more uncertain in comparison to the others.

Of the six taxa that responded to salinity, only *Pseudopedinella* spp. had a clear negative effect of salinity. Previous studies have detected an increase in the taxon, as well as a negative correlation with salinity (Suikkanen *et al.,* 2007). As measured in laboratory conditions by Ostroff *et al.* (1980), *P. pyriforme* has a relatively narrow salinity tolerance at 10°C, which is close to the 11.3°C it experiences in the area. Their study showed that the doublin time per day at salinities of both 2.5 and 7.5 was consistently lower than at salinity of 5. As all other observed effects were positive, the results would suggest that these taxa have their salinity optima at higher salinities. Notably, if salinity decreases in the Baltic Sea, the salinity optima of these, and perhaps other species would move further away from the mean conditions, possibly making the positive effect of salinity even stronger in the future. The consistently opposite signs of the salinity effect and the partial trend may either be coincidence or be a result of unobserved gradual changes, which have indirect effects on the biomasses of focal taxa. One mechanism could be salinity-induced change in the whole community, and hence, altered biotic interactions (Suikkanen *et al.,* 2013). Community shifts have been observed in phytoplankton communities in the Bothnian Sea and Bothnian Bay area (Kuosa *et al.,* 2017).

The diatom *Thalassiosira baltica,* which forms part of the spring bloom, was negatively influenced by the number of days with ice cover during the preceding winter. This result contrasts with previous studies, which have shown that diatoms are positively influenced by winter harshness, i.e. thick and long-lasting ice-cover (Kononen and Niemi, 1984; Klais *et al.,* 2011, 2013). Thus, our result implies that the response of *T. baltica* may differ from the common class-level response of diatoms. This is possible since *T. baltica* is only one of the common spring bloom diatom species in the study area, in addition to e.g. *Pauliella taeniata*, *S. marinoi* and *Chaetoceros* spp. (Andersson *et al.,* 2017). Concerning class-level biomass of diatoms, the ice break-up in spring leading to convective mixing should benefit diatoms that thrive in turbulent conditions (Wasmund *et al.,* 2013), but the possible dominance of diatoms over dinoflagellates during the spring bloom is mainly caused by conditions well before the establishment of thermal stratification (Kremp *et al.,* 2008).

We observed mostly negative stratification effects among our studied taxa, suggesting these would benefit from more turbulent conditions. This concerned the colonial cyanobacteria *Snowella* and *Woronichinia* spp. and some of the smaller flagellates such as *Hemiselmis* spp., and *Teleaulax* spp. The cyanobacteria *Snowella* and *Woronichinia* spp. have previously been associated with colder, more turbulent periods of the year (Laamanen, 1997).

By the end of the century, the salinity in the Gulf of Finland is expected to decrease with 1–2 units and the temperature to increase by 2–3°C due to e.g. climate change (Meier *et al.,* 2012). In the taxa with observed effects (Table II) these changes correspond to approximately a 3.9–7.8 SD decrease in salinity and a 2.3–3.4 SD rise in temperature. For example, in *Dinobryon faculiferum* and *Protoperidinium bipes*, which showed clear positive effects of salinity, our model would predict a ca 84–98% decrease in biomass. Most of the taxa showing effects were linked to either salinity, temperature or stratification, and future changes could cause substantial effects on these taxa. Compared to the variability in temperature and salinity, which has been experienced by the taxa so far (Table II), a 1 or 2 unit change in either will be substantial. In our study, the variability in salinity and temperature ranges mostly did not cover the values, which might be observed in the future (Meier *et al.,* 2012).

One of the goals of the present study was to use specific species- or genus-level data, since previous studies have suggested that pooling biomasses to class level can influence the results (Griffiths *et al.,* 2016). For example, different cyanobacterial taxa (class Cyanophyceae) responded differently to temperature; the temperature effect on the annual biomass of *Snowella* spp. was negative, whereas it was somewhat positive on the biomass of *Aphanizomenon* spp. and *Woronichinia* spp. Also, stratification seemed to affect the annual biomasses of cyanobacteria *Snowella* spp. and *Woronichinia* spp., but not the biomasses of the other cyanobacteria. Different chrysophyte taxa (class Chrysophyceae) also responded differently to some environmental variables. For example, models of both *Pseudopedinella* spp. and *D. faculiferum* showed support for salinity effects, but the effect was not in the same direction. Using class level data might thus average out effects; this would be particularly pronounced in groups like the cyanobacteria where the contribution to the overall biomass varies substantially between taxa, e.g. *Aphanizomenon* spp. contribute with ~ 30% to annual biomass, while *Snowella* spp. contributes 0.7%.

The present study only looked at abiotic predictors. In a community study Barraquand *et al.* (2018) highlighted the importance of top-down control in phytoplankton as suggested by high intra-specific density dependence coupled with few positive inter-group interactions. We did not investigate any community interactions, but out of the 14 taxa with no environmental effects, only the green algae and *Planktolyngbya* exhibited other than compensatory dynamics (undercompensatory). We also detected only one nutrient effect, but the nutrient observations were temporally further away from the relative biomass estimate and not tailored to the timing of the biomass increase.

### The day-level observation model

As our study focused on annual level estimates, it was ideal to remove day-level noise, and we considered that wind could affect the observation error by altering sampling efficiency. It is, however, relevant to note that wind can drive short-term within-season population dynamics in these short-lived and rapidly reproducing organisms (Kanoshina *et al.,* 2003; Lips and Lips, 2010). Hence, many short-term population fluctuations following quick recovery may here be assigned to the observation model, rather than to the annual-level population model. This affects the interpretation of observation errors and population dynamic processes at the current scale of investigation. When the focus is on annual biomass dynamics, short-term fluctuations can be justified as noise, while prolonged periods of suitable or adverse conditions may affect total biomasses for a given year.

Wind speed affected the biomass of some of the taxa, which is not surprising as the area is very exposed and prone to wind action; Majaneva *et al.* (2009) suggested that the biomass variance was higher at two exposed sampling stations compared to at a sheltered one. In samples collected with the ferrybox method, the annual biomasses were consistently lower for seven taxa, but we could not find any common reason for this, since these taxa represented different taxonomic classes, cell volumes, and modes of motility or buoyancy. Increased wind could lead to redistribution of biomass for taxa like *Nodularia* spp., which tends to aggregate at the surface, and thus make sampling more efficient as the surface is challenging to sample (Kanoshina *et al.,* 2003). On the other hand, ferrybox samples might have a different effect as the samples are taken at a depth of 4 or 5 m, onboard a ship en route, while integrated samples are taken from top 0 to 10 m. In this case, the ship itself might have a bigger impact than any wind induced mixing. For other taxa, such as the euglenophyte *Eutreptiella* spp. that has its biomass maximum usually below the typical 10 m sampling depth (Olli *et al.,* 1996), the results could be positively biased during strong winds that redistribute deeper aggregates and transfer the species upwards within sampling range. Cryptophytes *Teleaulax* spp. and *Hemiselmis* spp., which also both showed positive responses to wind, have been shown to aggregate below our sampling depth of 10 m (Hajdu *et al.,* 2007). The prymnesiophyte *Chrysochromulina* spp., however, concentrates in the upper 10 m according to Hajdu *et al.* (2007). Hence, the negative wind effect on *Chrysochromulina* spp. might be due to a displacement below the maximum 10 m sampling depth.

The observation model corrected for the variable timing of the sampling events, by assuming a consistent pattern of phenology for each taxa annually, and for the influence of wind on the sampled biomass. Our aim was to obtain a measure that considered the annually repeated pattern in biomass dynamics that many plankton taxa exhibit. This is not a perfect approach, as the observation model (GAMM) does not account for variable biomass phenology (e.g. timing of mean biomass) between years, and we noticed some discrepancies in our diagnostics. These discrepancies in the form of temporally correlated positive outliers suggest that there could have been a change in phenology in nine of our taxa over the investigated time-period. In the Baltic Sea, shifts in phenology have been reported leading to earlier spring blooms (Klais *et al.,* 2011; Lips *et al.,* 2014; Hjerne *et al.,* 2019) and cyanobacterial blooms (Kahru and Elmgren, 2014). The potential shift in timing of biomass succession could have led to added noise in our annual biomass estimates, subsequently influencing the detection of environmental effects in the SSM analysis. Looking into specific phenology effects is not within the scope of this paper, but it could be worthwhile to study phenology in closer detail as other areas have also reported effects on plankton (Edwards and Richardson, 2004) and considered the interplay of phenology and environmental conditions (Scharfe and Wiltshire, 2019). A shift in timing can affect the results of long-term trends if sampling is carried out over a narrow time-window. As many plankton taxa have complicated life histories with multiple annual peaks, one might end up in a situation where the same peak is not measured every year (Scharfe and Wiltshire, 2019).

## CONCLUSION

We used decadal monitoring data and a SSM approach to detect extrinsic drivers of annual relative species- or genus-specific plankton biomasses. The annual dynamics in median biomass were mostly related to temperature, salinity and stratification. The taxa experienced relatively little annual variation in salinity during 2 months prior to median biomass timing, which suggest that they could be sensitive to future climate change scenarios predicting reductions in salinity of 1–2 units. Rising temperatures are expected to benefit taxa that were positively influenced by temperature, including, such as bloom forming *Aphanizomenon* spp. and the potentially toxic *D. acuminata* (Kamiyama *et al.,* 2010). We detected especially strong negative effects of ice on the biomass of *T. baltica*, contradicting previous findings on diatoms. Investigating the species- and genus-level, we found within-class differences, both in the variables identified as relevant and in the directions of observed effects of certain variables. Additionally, we detected a systematic pattern in the residuals in the day-level model, which suggests that a phenological shift might have occurred in nine of the investigated taxa. Further studies are needed to confirm this possible shift and its magnitude.

## Supplementary Material

Forsblom_et_al_supplementary-material_fbz063
