# Supplementary material for: Environmental variables driving species and genus level changes in annual plankton biomass
Source: J Plankton Res. 2019 Dec 19;41(6):925–38. doi: 10.1093/plankt/fbz063 (PMC6946087; doi:10.1093/plankt/fbz063)
Supplement: Forsblom_et_al_supplementary-material_fbz063 [file forsblom_et_al_supplementary-material_fbz063.pdf]

Supplementary Material

Environmental variables driving species and genus level changes in annual plankton biomass

Louise Forsblom, Jonna Engström-Öst, Sirpa Lehtinen, Inga Lips, and Andreas Lindén

This document contains the following supplementary material:

p. 2	List of competing models assessed through model selection
p. 3	Method description concerning annual taxon-specific temperature, salinity and stratification anomalies.
p. 4	Supplementary Figure 1
p. 4	Supplementary Figure 2
p. 5–7	Supplementary Figure 3
p. 8–10	Supplementary Figure 4
p. 11–13	Supplementary Figure 5
p. 14	Supplementary Figure 6
p. 15–16	Supplementary Figure 7
p. 17	Supplementary Table I
p. 18	Supplementary Table II
p. 19–22	Supplementary Table III
p. 23	Supplementary Table IV

List of competing models assessed through model selection

Models considered for all taxa:

year + temperature
year + salinity
year + stratification index

Models additionally considered for taxa that reach median biomass prior to 15 June:

year + ice
year + DIN
year + DIP

Models additionally considered for diatoms and *Ebria tripartita:*

year + silicate

Taxon-specific environmental covariates for the annual model

We considered effects of salinity, temperature and stratification index as covariates for all taxa in the state-space models describing inter-annual biomass dynamics. These annual covariates were constructed by first fitting a statistical model to the raw data.

We used monitoring data on temperature and salinity, averaged for the top ten meters, as well as the calculated stratification index (Equation 1 in article) from all seasons. The data were retrieved in two parts from the data portal of The International Council for the Exploration of the Sea 29.10.2018. The area was restricted to be 22.84 to 26.40 longitude and 59.28 to 60.20 latitude. Stations were further filtered to have a depth of at least 40 meters, and to further restrict the data to pelagic stations twelve coastal stations were manually removed.

For each taxon, observations were used from a 60-day wide time window, ending on the day when the taxon on average reaches its median biomass. The taxon-specific median dates (when 50% of the cumulative biomass was reached) were calculated from the fitted smoothing functions for Julian day in the day-level observation models for daily biomasses (see Methods in manuscript). The timing of the median is reported in Table I in the article. This was necessary, as the goal was to capture the environmental covariates that affected the period of most substantial accumulation of the biomass.

Each of the three variables were individually analysed as the response variable in generalised additive mixed models (GAMM), over unique time-windows for each taxon separately. The GAMM applied had cyclic smoothing functions of Julian day and random effects (on the intercept) of factors year and location. We further applied identity link functions and Gaussian error distributions. Time series of the annual mean anomalies for temperature, stratification index, and salinity were subsequently created by extracting the fitted random effects of factor variable year. The final time series of covariates applied were obtained by z-scoring the annual random effect estimates. The taxon-wise results are presented in Figures S3, S4 and S5.

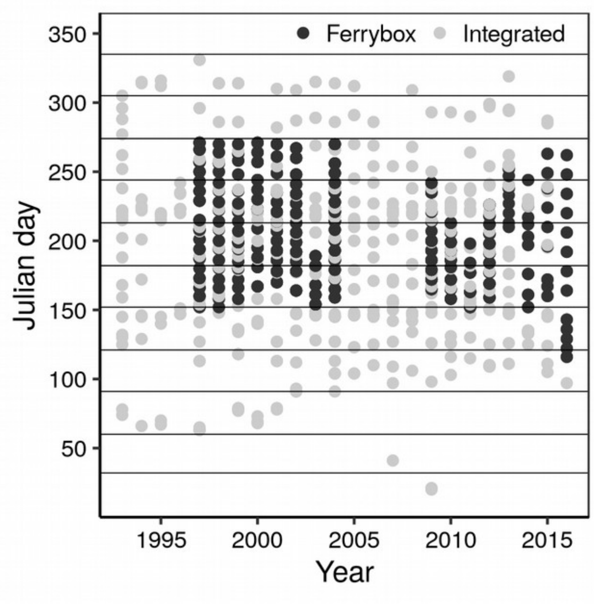

Fig. S1. The temporal pattern of the sampling events with the Julian Day of all the sampling events for all years. First day of each month marked with horizontal line. Sampling with ferrybox in black and integrated sampling in grey.

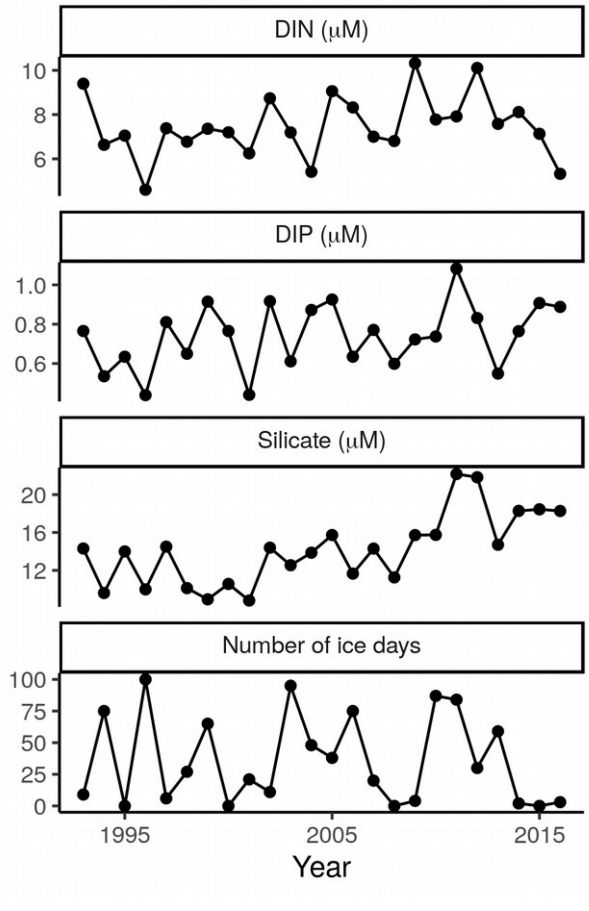

Fig. S2. Time series of nutrient and ice-conditions presented with their original units. DIN, DIP, and silicate are annual wintertime means, and ice is the annual number of days with ice-cover. In contrast to temperature, salinity and stratification, these variables are the same for all taxa they were applied to. However, notice that all covariates were z-scored before the actual analyses.

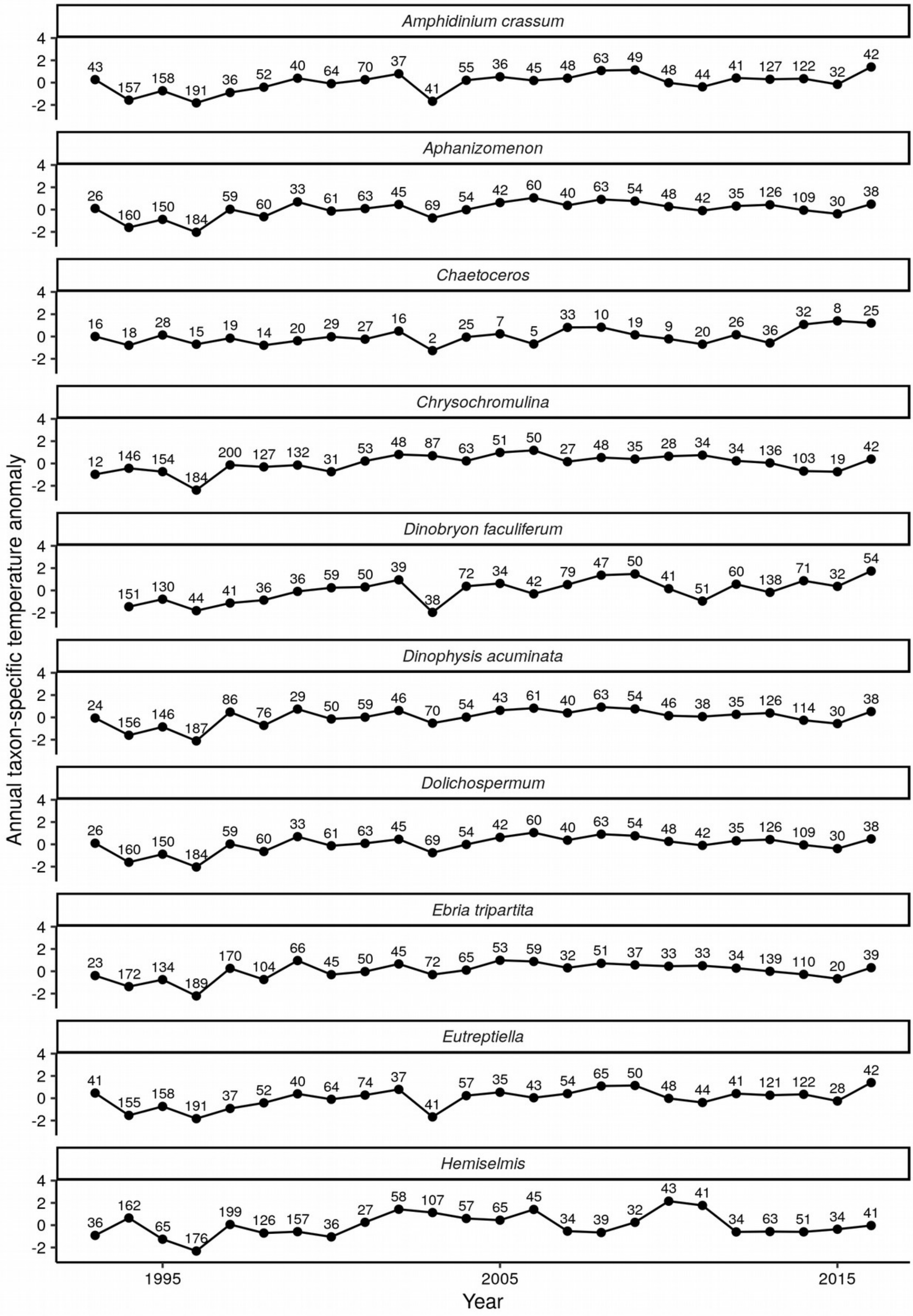
Amphidinium crassum
43 157 158 191 36 52 40 64 70 37 41 55 36 45 48 63 49 48 44 41 127 122 32 42
Aphanizomenon
26 160 150 184 59 60 33 61 63 45 69 54 42 60 40 63 54 48 42 35 126 109 30 38
Chaetoceros
16 18 28 15 19 14 20 29 27 16 2 25 7 5 33 10 19 9 20 26 36 32 8 25
Chrysochromulina
12 146 154 184 200 127 132 31 53 48 87 63 51 50 27 48 35 28 34 34 136 103 19 42
Dinobryon faculiferum
151 130 44 41 36 36 59 50 39 38 72 34 42 79 47 50 41 51 60 138 71 32 54
Dinophysis acuminata
24 156 146 187 86 76 29 50 59 46 70 54 43 61 40 63 54 46 38 35 126 114 30 38
Dolichospermum
26 160 150 184 59 60 33 61 63 45 69 54 42 60 40 63 54 48 42 35 126 109 30 38
Ebria tripartita
23 172 134 189 170 104 66 45 50 45 72 65 53 59 32 51 37 33 33 34 139 110 20 39
Eutreptiella
41 155 158 191 37 52 40 64 74 37 41 57 35 43 54 65 50 48 44 41 121 122 28 42
Hemiselmis
36 162 65 176 199 126 157 36 27 58 107 57 65 45 34 39 32 43 41 34 63 51 34 41
4
2
0
-2
1995
2005
2015
Year
Annual taxon-specific temperature anomaly

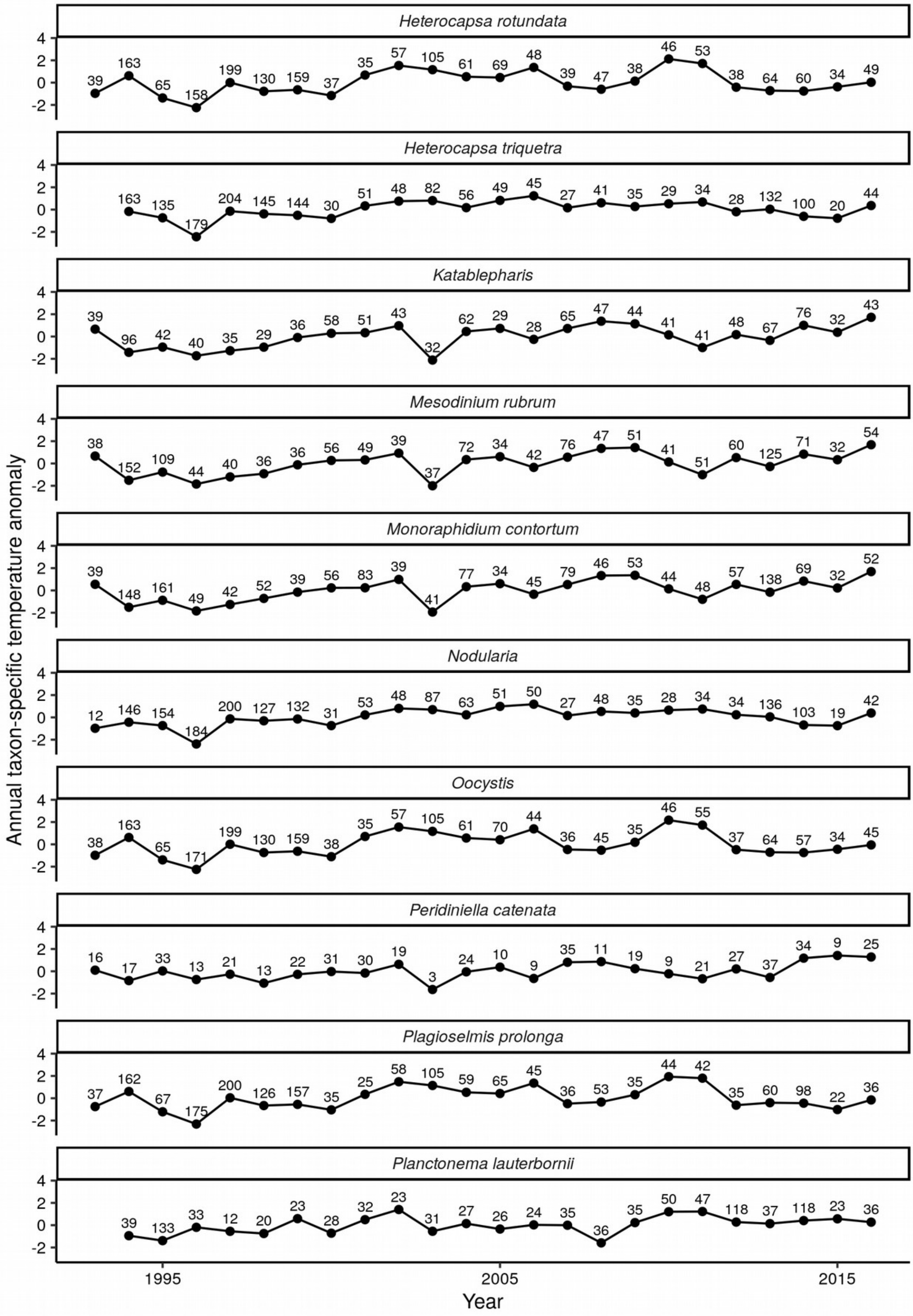
Heterocapsa rotundata
39 163 65 158 199 130 159 37 35 57 105 61 69 48 39 47 38 46 53 38 64 60 34 49
Heterocapsa triquetra
163 135 179 204 145 144 30 51 48 82 56 49 45 27 41 35 29 34 28 132 100 20 44
Katablepharis
39 96 42 40 35 29 36 58 51 43 32 62 29 28 65 47 44 41 41 48 67 76 32 43
Mesodinium rubrum
38 152 109 44 40 36 36 56 49 39 37 72 34 42 76 47 51 41 51 60 125 71 32 54
Monoraphidium contortum
39 148 161 49 42 52 39 56 83 39 41 77 34 45 79 46 53 44 48 57 138 69 32 52
Nodularia
12 146 154 184 200 127 132 31 53 48 87 63 51 50 27 48 35 28 34 34 136 103 19 42
Oocystis
38 163 65 171 199 130 159 38 35 57 105 61 70 44 36 45 35 46 55 37 64 57 34 45
Peridiniella catenata
16 17 33 13 21 13 22 31 30 19 3 24 10 9 35 11 19 9 21 27 37 34 9 25
Plagioselmis prolonga
37 162 67 175 200 126 157 35 25 58 105 59 65 45 36 53 35 44 42 35 60 98 22 36
Planctonema lauterbornii
39 133 33 12 20 23 28 32 23 31 27 26 24 35 36 35 50 47 118 37 118 23 36
4
2
0
-2
Annual taxon-specific temperature anomaly
1995
2005
2015
Year

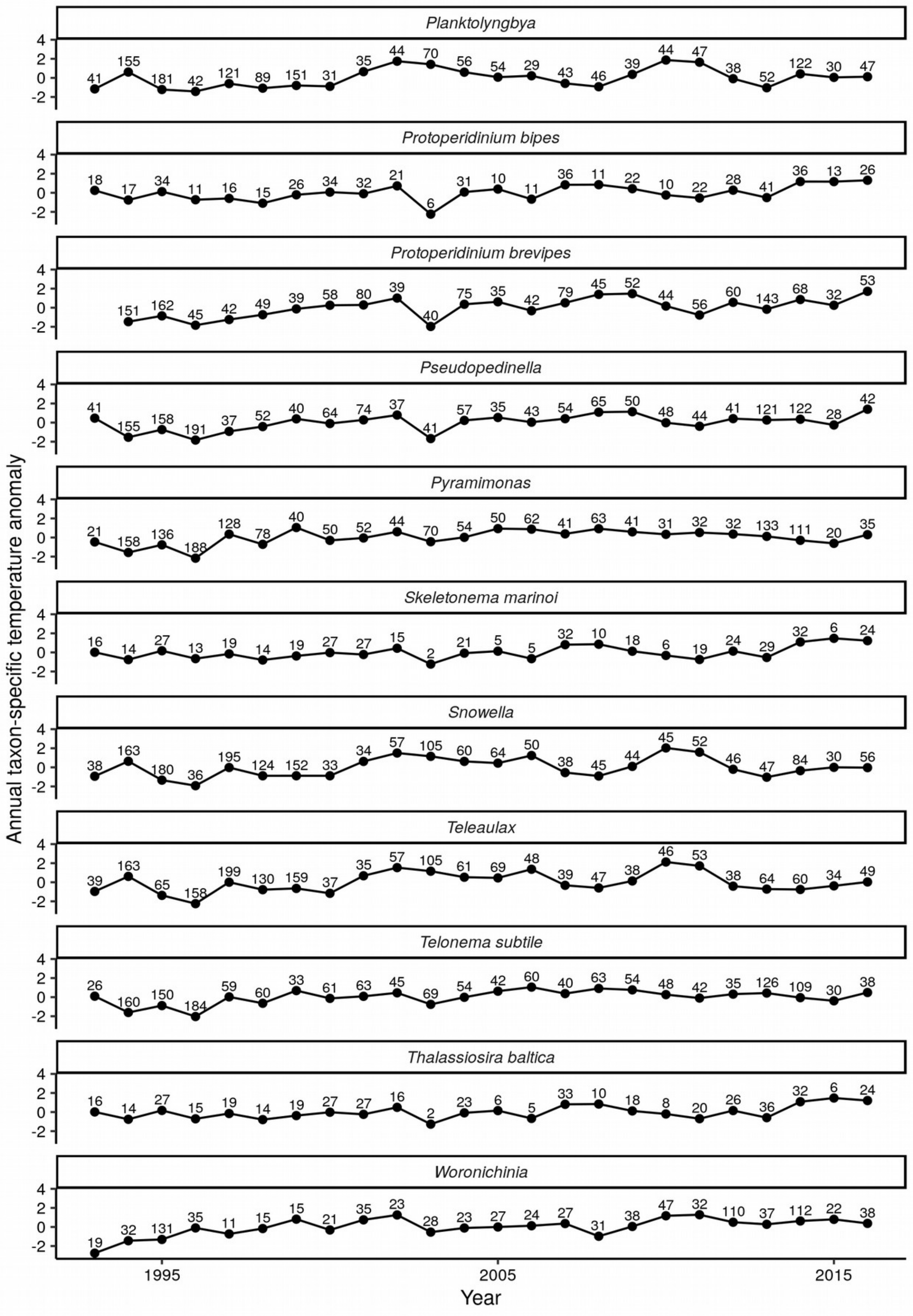

Fig. S3. The annual anomaly from the mean temperature during 0–60 days prior to median biomass occurrence for each taxon.

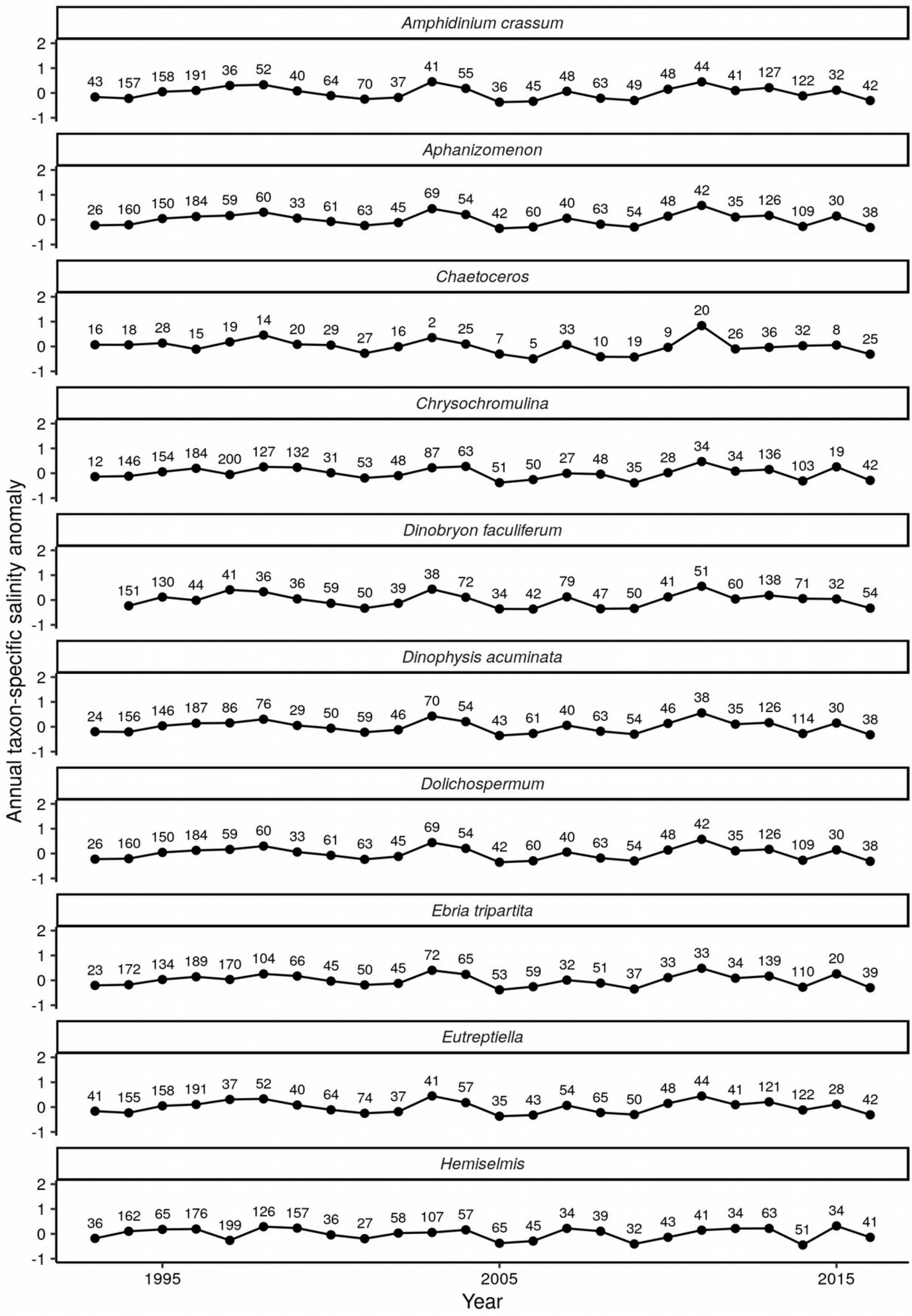

Amphidinium crassum
Aphanizomenon
Chaetoceros
Chrysochromulina
Dinobryon faculiferum
Dinophysis acuminata
Dolichospermum
Ebria tripartita
Eutreptiella
Hemiselmis
Annual taxon-specific salinity anomaly
Year
1995
2005
2015

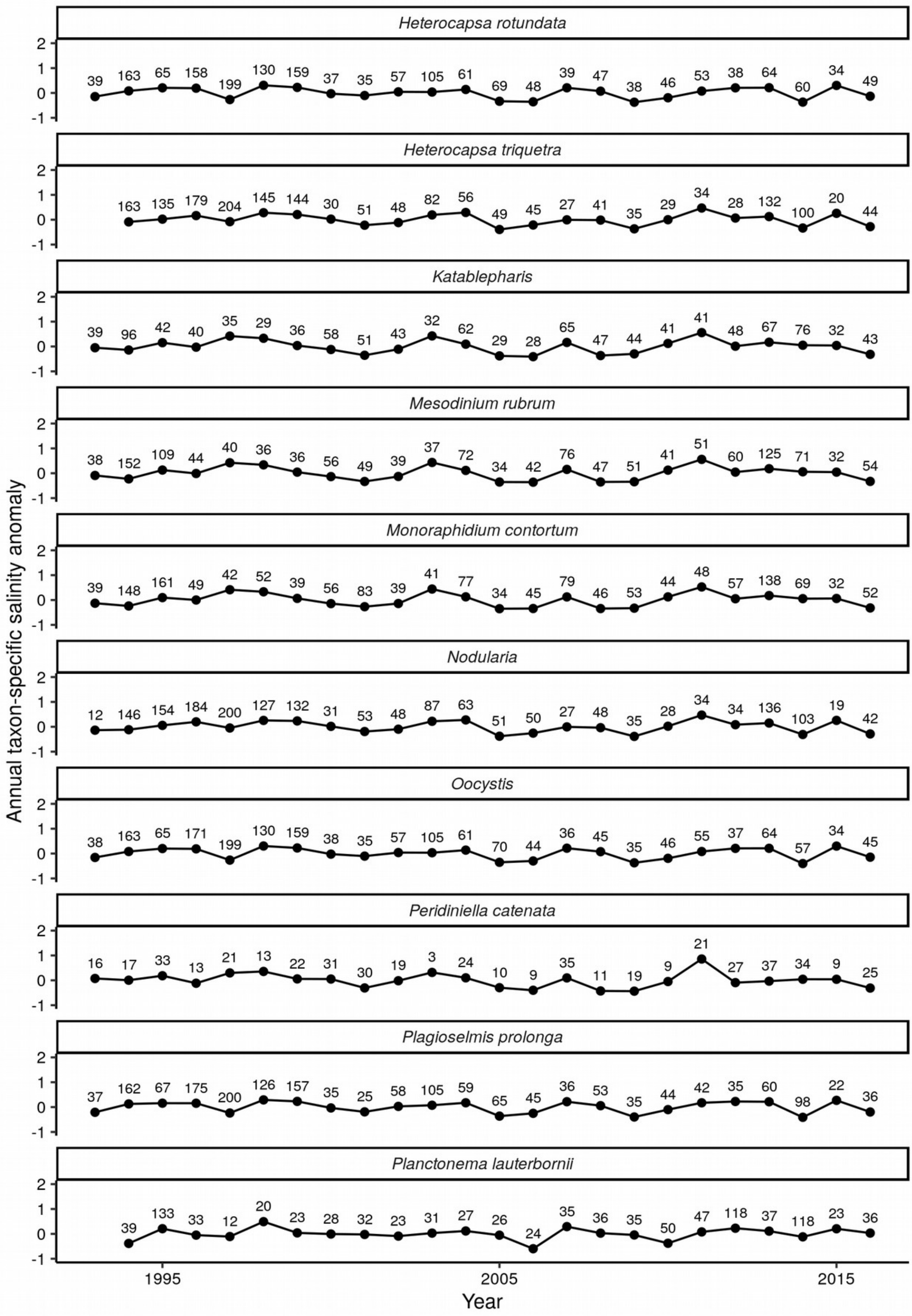
Heterocapsa rotundata
Heterocapsa triquetra
Katablepharis
Mesodinium rubrum
Monoraphidium contortum
Nodularia
Oocystis
Peridiniella catenata
Plagioselmis prolonga
Planctonema lauterbornii
Annual taxon-specific salinity anomaly
Year
1995
2005
2015

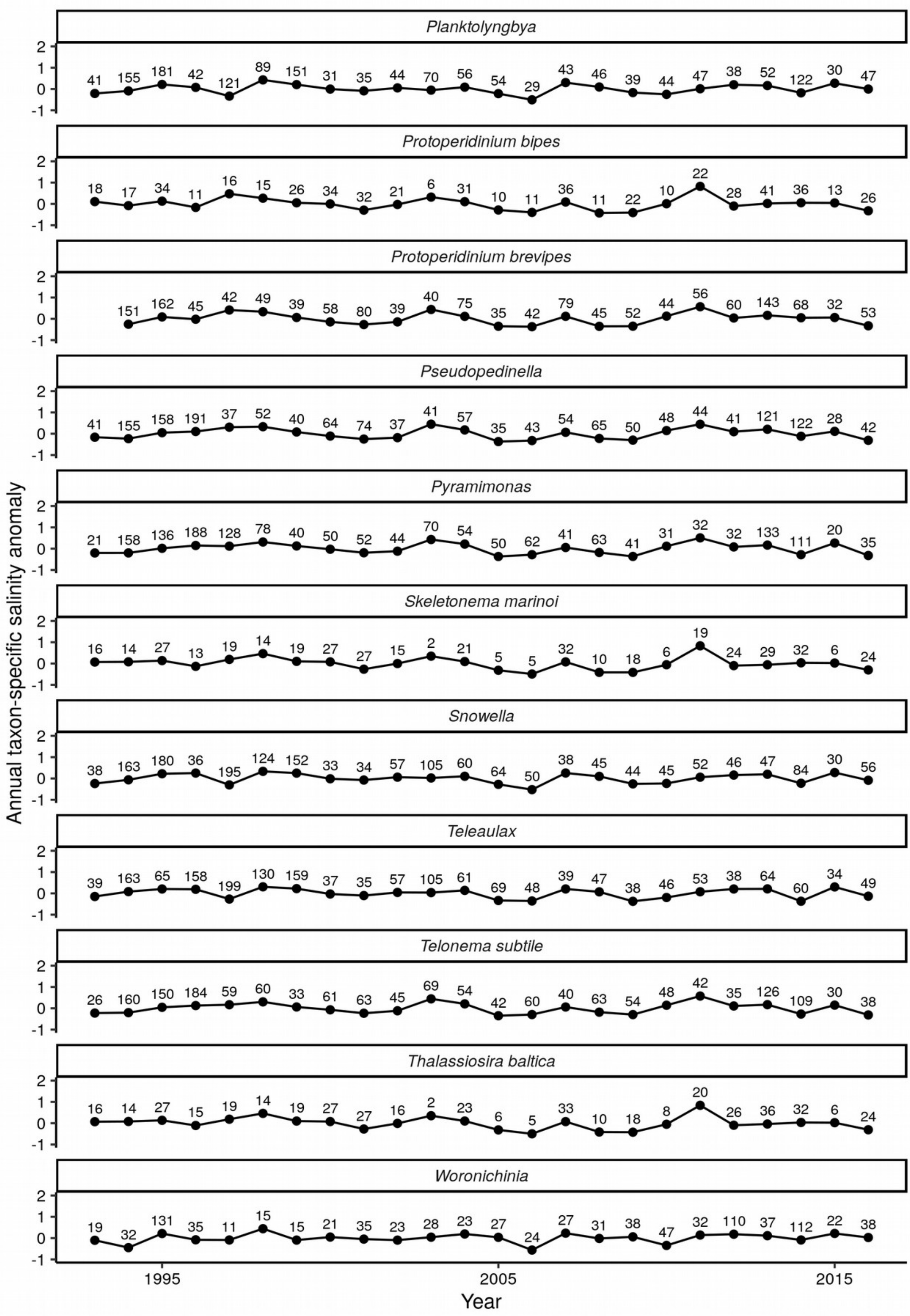

Fig. S4. The annual anomaly from the mean salinity during 0–60 days prior to median biomass occurrence for each taxon.

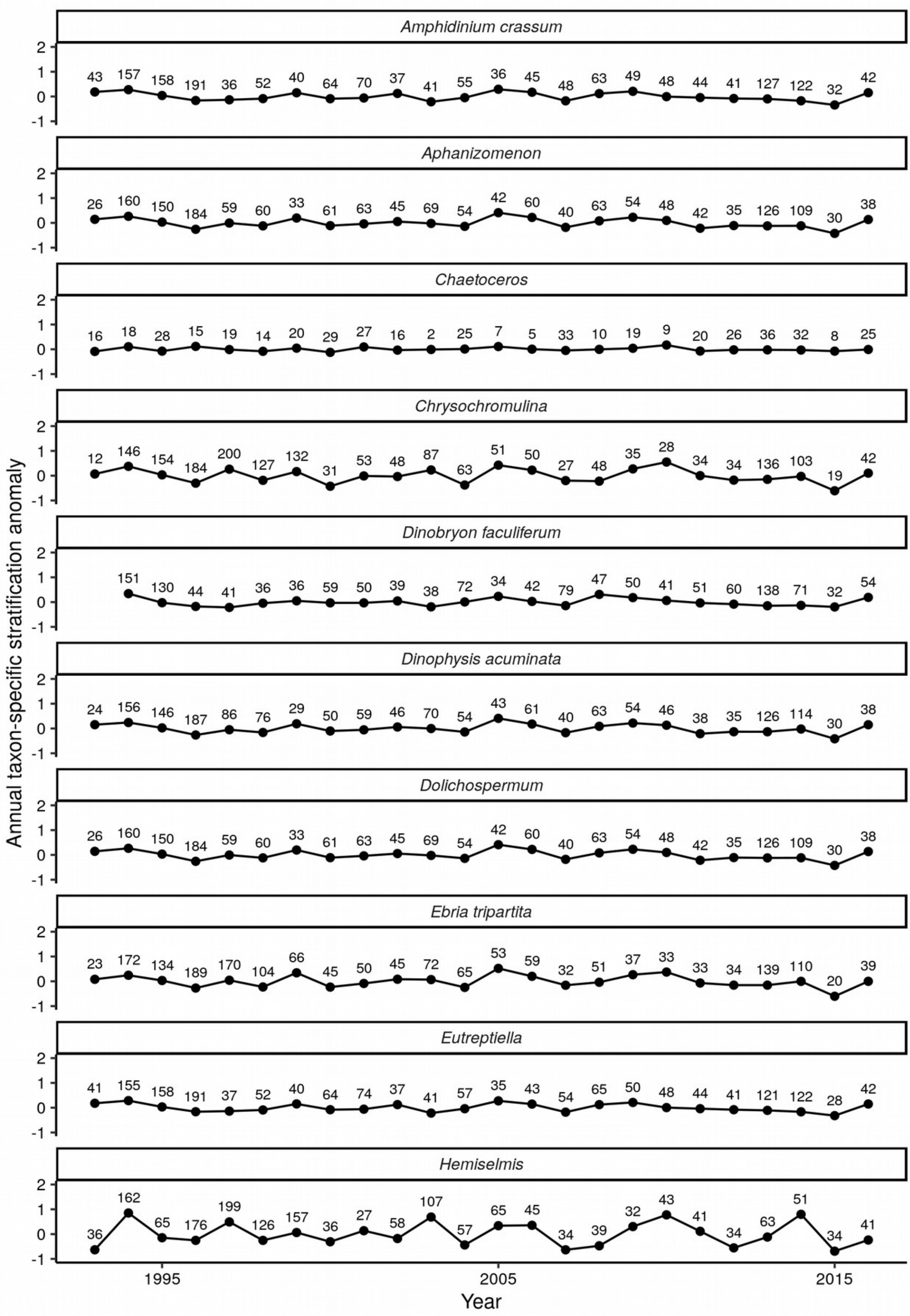
Amphidinium crassum
Aphanizomenon
Chaetoceros
Chrysochromulina
Dinobryon faculiferum
Dinophysis acuminata
Dolichospermum
Ebria tripartita
Eutreptiella
Hemiselmis
Annual taxon-specific stratification anomaly
Year
1995
2005
2015

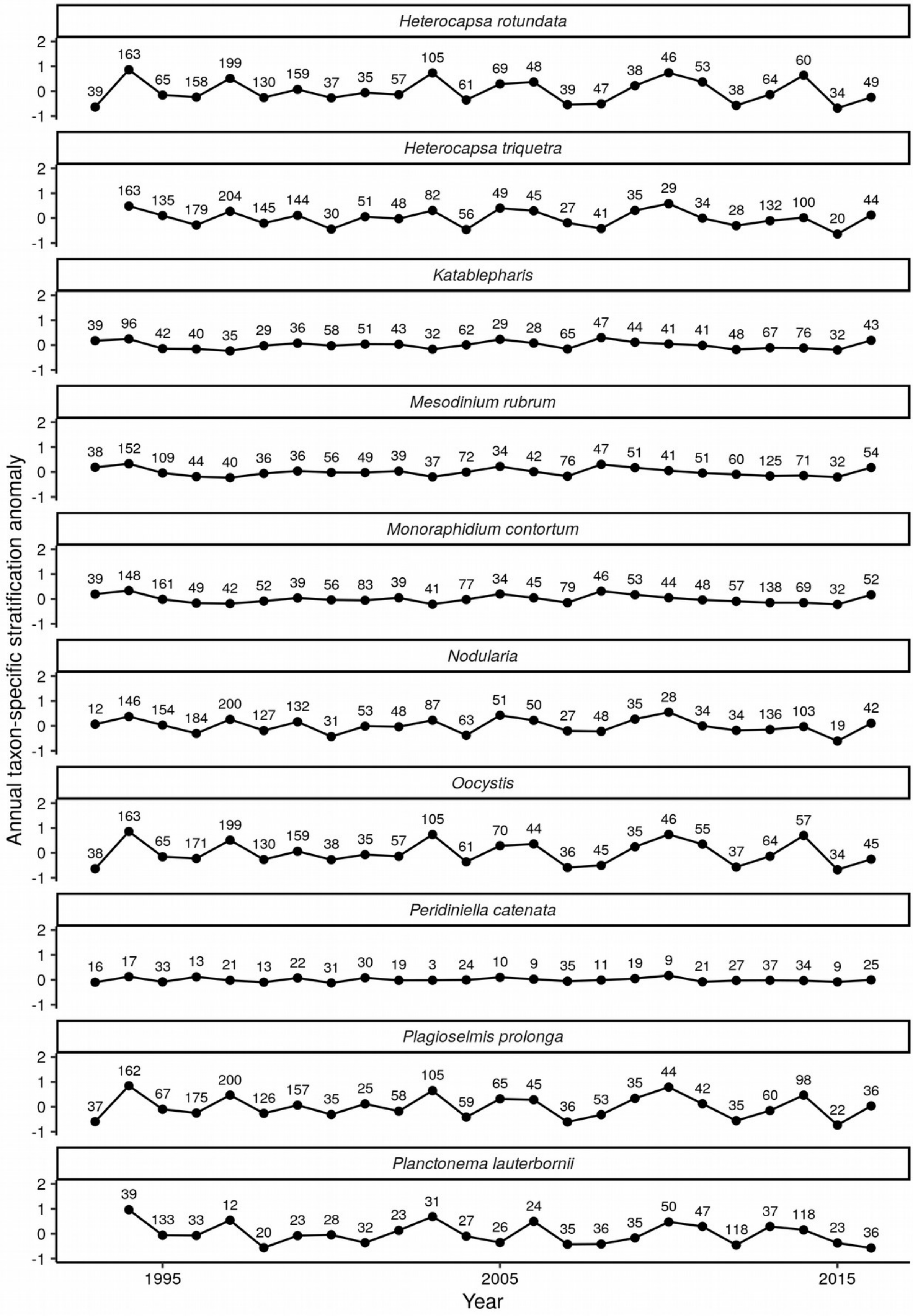
Heterocapsa rotundata
Heterocapsa triquetra
Katablepharis
Mesodinium rubrum
Monoraphidium contortum
Nodularia
Oocystis
Peridiniella catenata
Plagioselmis prolonga
Planctonema lauterbornii
Annual taxon-specific stratification anomaly
Year
1995
2005
2015

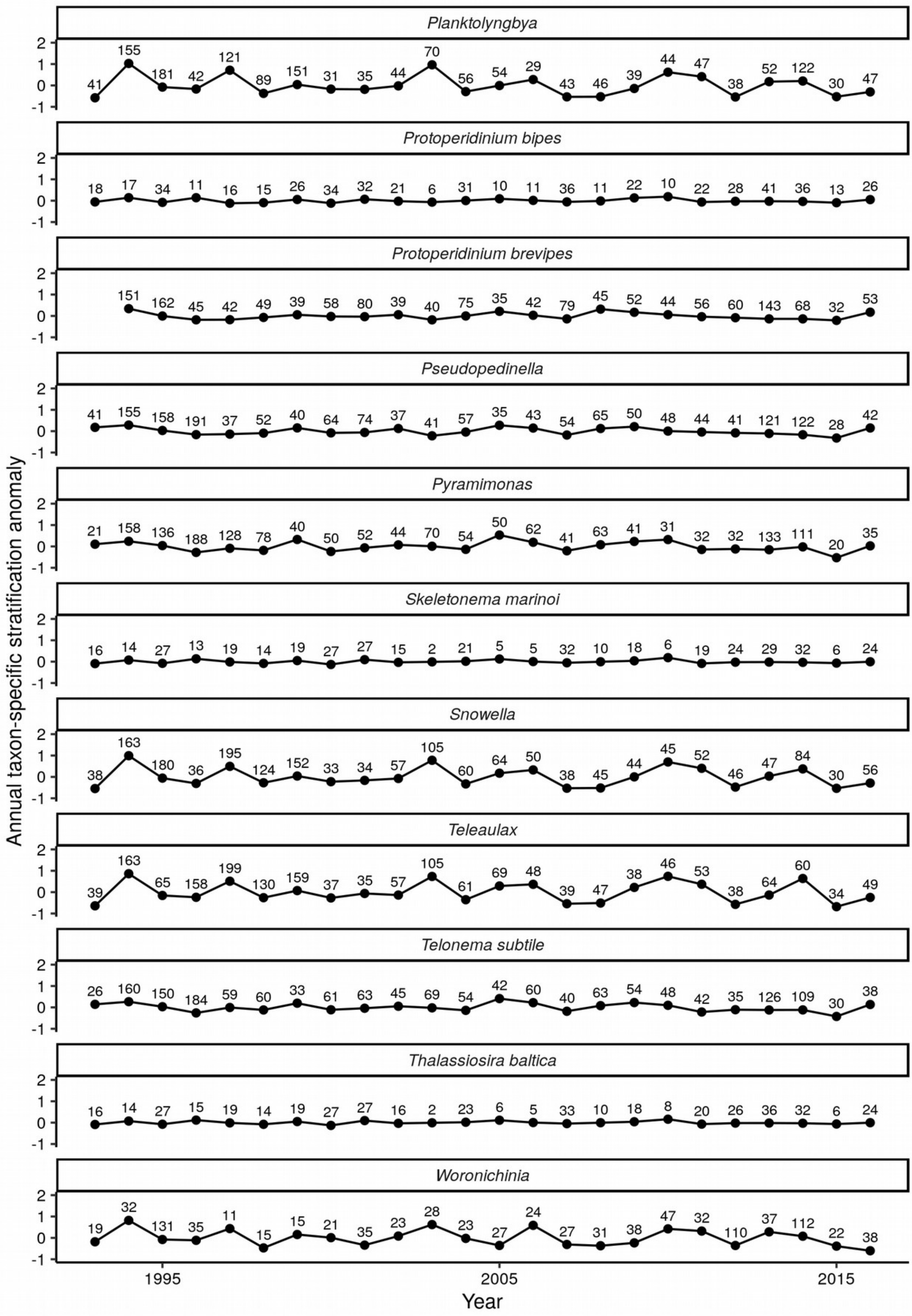

Fig. S5. The annual anomaly from the mean stratification index during 0–60 days prior to median biomass occurrence for each taxon.

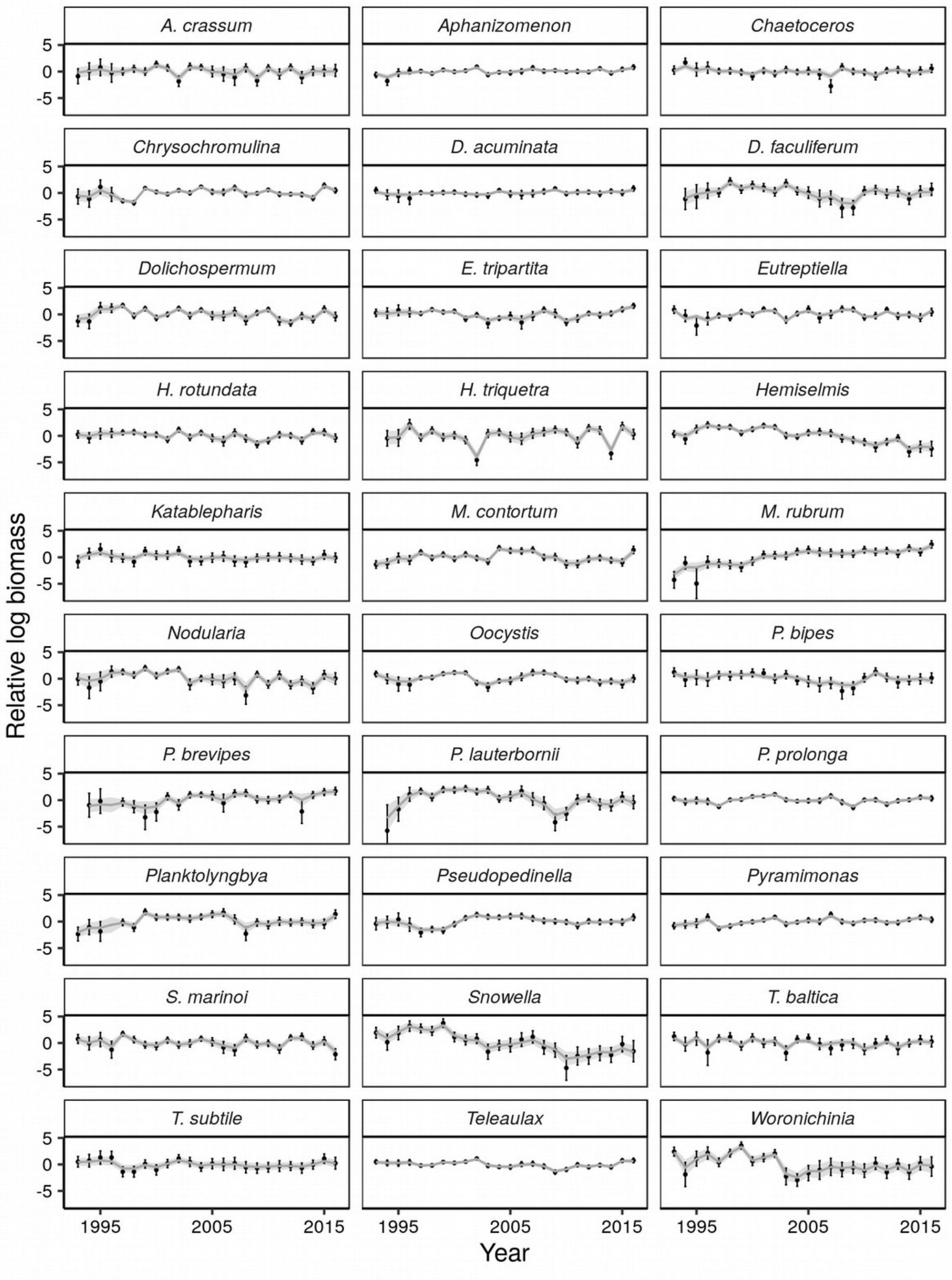

Fig. S6. Time series from the annual analysis (line) together with 95% CI (light grey area). Shown together with the relative annual biomass estimates for each taxon of interest, as constructed by the GAMM analysis (points, error bars correspond to ± 1.96 SE) .

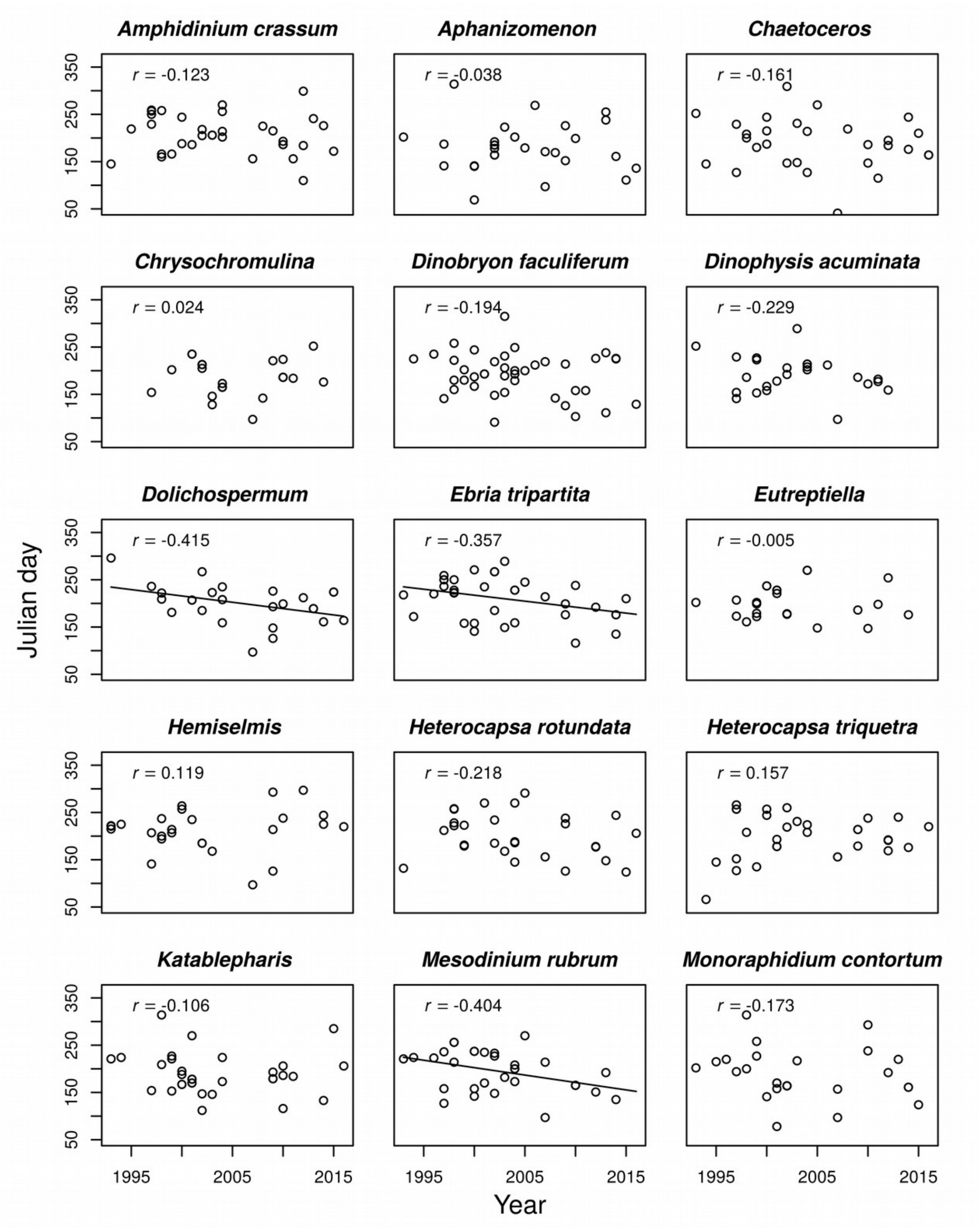

Fig. S7. Scatter plots with Pearson correlation of Dunn–Smyth residuals of the most extreme data points (SD > 2) with year against Julian day for all taxa. Regression lines have been drawn for taxa where the correlation was significant ($p < 0.05$). Graph continues on the next page.

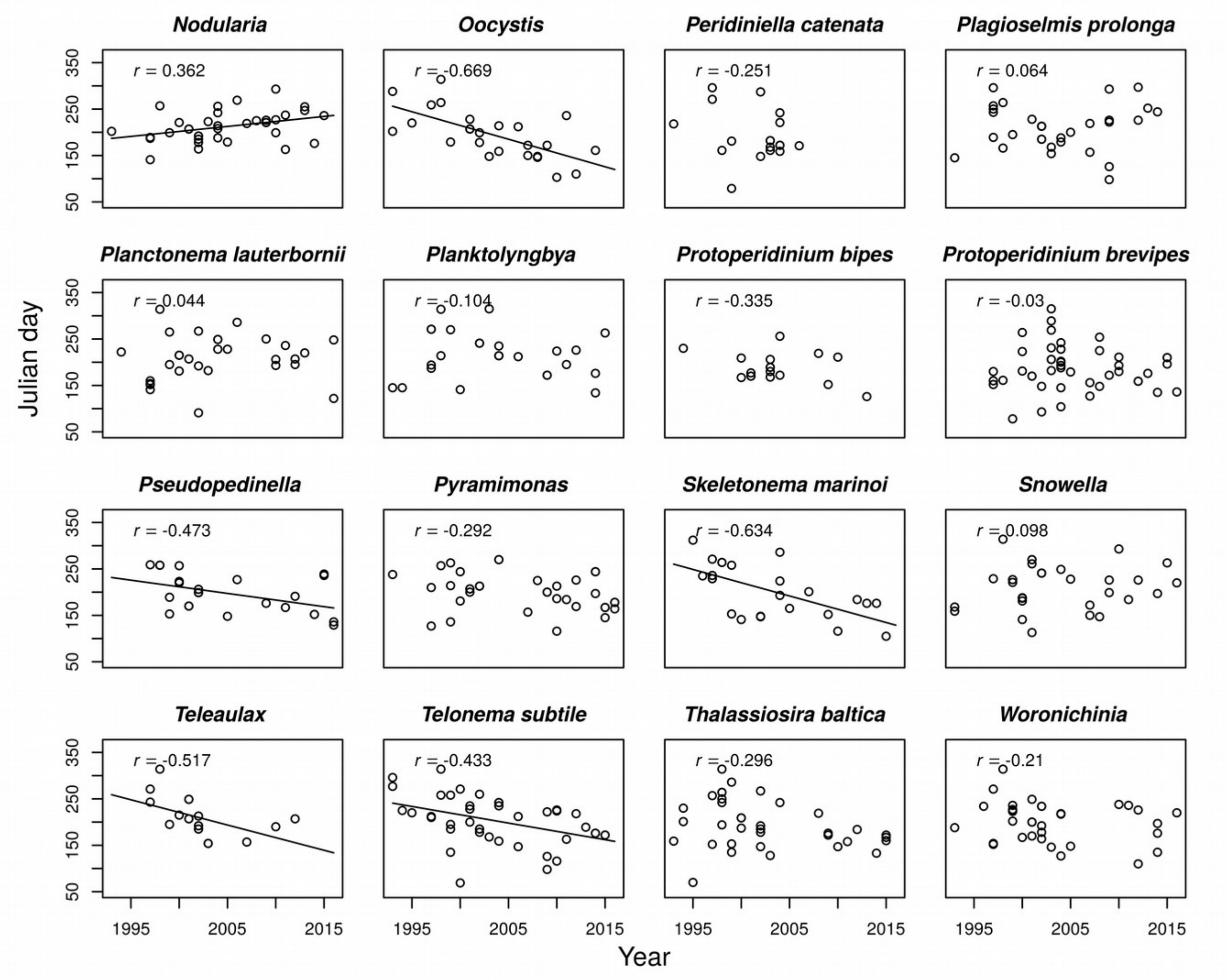

Fig. S7 continued.

Supplementary tables SI, SII, SIII and SIV

Table SI: Additional information on the phytoplankton samples included in the analysis, including the station names (Station), sampling method (Method), data source (institute), approximate coordinates, temporal span and number of samples (N). (Institute coded: EMI = Estonian Marine Institute, University of Tartu; SYKE = Finnish Environment Institute; MSI = Marine Systems Institute, Tallinn University of Technology).

Station	Method	Source	Latitude	Longitude	First year	Last year	*N*
14	Integrated sampling	EMI	59.83	25.62	2014	2014	2
17	Integrated sampling	EMI	59.72	25.02	2008	2014	8
18	Integrated sampling	EMI	59.63	25.18	2008	2014	8
19	Integrated sampling	EMI	59.61	24.35	2014	2014	2
2	Integrated sampling	EMI	59.54	24.69	1996	2013	191
23	Integrated sampling	EMI	59.33	23.27	2011	2014	3
E80	Integrated sampling	EMI	59.71	25.71	2014	2014	2
F1	Integrated sampling	EMI	59.92	26.34	1996	2014	34
F3	Integrated sampling	EMI	59.84	24.84	1996	2015	122
H1	Integrated sampling	EMI	59.45	22.95	1996	2015	50
LL12	Integrated sampling	SYKE	59.48	22.90	1993	2016	21
LL12A	Integrated sampling	SYKE	59.60	22.90	1993	2013	1
LL3	Integrated sampling	SYKE	60.15	26.33	1993	2013	1
LL3A	Integrated sampling	SYKE	60.07	26.35	1993	2014	20
LL7	Integrated sampling	SYKE	59.85	24.84	1993	2015	26
GXY10	Ferrybox	MSI	59.63	24.67	2009	2012	29
GXY11	Ferrybox	MSI	59.71	24.66	2011	2011	4
GXY12	Ferrybox	MSI	59.67	24.66	2009	2010	19
GXY13	Ferrybox	MSI	59.64	24.66	2011	2011	4
GXY14	Ferrybox	MSI	59.58	24.68	2009	2010	19
GXY4	Ferrybox	MSI	59.86	24.79	2012	2014	13
GXY5	Ferrybox	MSI	59.93	24.85	2011	2011	3
GXY6	Ferrybox	MSI	59.92	24.84	2009	2016	60
GXY7	Ferrybox	MSI	59.86	24.79	2011	2011	4
GXY8	Ferrybox	MSI	59.67	24.66	2009	2016	60
GXY9	Ferrybox	MSI	59.79	24.72	2011	2011	3
TH2	Ferrybox	MSI	59.69	24.64	2013	2013	8
TH3	Ferrybox	MSI	59.80	24.74	2013	2013	3
WQ6	Ferrybox	EMI	59.93	24.93	1997	2004	71
WQ7	Ferrybox	EMI	59.83	24.80	1997	2004	110
WQ8	Ferrybox	EMI	59.72	24.62	1997	2004	78
WQ9	Ferrybox	EMI	59.62	24.68	1997	2004	110

Table II: The standard deviations used in the z-scoring of all the environmental covariates for the investigated taxa (n = number of years, Temp = temperature, Sal = salinity, Strat = stratification index). The taxon-specific mean values for temperature salinity and stratification can be found in table II in the manuscript. The mean values for the rest of the covariates for 23 or 24 years respectively are: 7.39 or 7.48 for DIN, 0.74 or 0.74 for DIP, 14.15 and 14.15 for SiO_4*, 37.0 or 35.8 for Ice, and 12 or 12.5 for Year.*

Taxon	*n*	Temp	Sal	Strat	DIN	DIP	SiO_4	Ice	Year
Amphidinium crassum	24	0.85	0.25	0.17	1.40	0.17	3.76	35.11	7.07
Aphanizomenon spp.	24	0.75	0.25	0.19	1.40	0.17	3.76	35.11	7.07
Chaetoceros spp.	24	0.70	0.30	0.08	1.40	0.17	3.76	35.11	7.07
Chrysochromulina spp.	24	0.79	0.23	0.29	1.40	0.17	3.76	35.11	7.07
Dinobryon faculiferum	23	1.03	0.28	0.16	1.37	0.17	3.84	35.42	6.78
Dinophysis acuminata	24	0.76	0.25	0.19	1.40	0.17	3.76	35.11	7.07
Dolichospermum spp.	24	0.75	0.25	0.19	1.40	0.17	3.76	35.11	7.07
Ebria tripartita	24	0.77	0.24	0.25	1.40	0.17	3.76	35.11	7.07
Eutreptiella spp.	24	0.85	0.25	0.17	1.40	0.17	3.76	35.11	7.07
Hemiselmis spp.	24	1.06	0.24	0.48	1.40	0.17	3.76	35.11	7.07
Heterocapsa rotundata	24	1.07	0.22	0.47	1.40	0.17	3.76	35.11	7.07
Heterocapsa triquetra	23	0.78	0.23	0.33	1.37	0.17	3.84	35.42	6.78
Katablepharis spp.	24	1.03	0.27	0.16	1.40	0.17	3.76	35.11	7.07
Mesodinium rubrum	24	1.02	0.27	0.16	1.40	0.17	3.76	35.11	7.07
Monoraphidium contortum	24	1.00	0.26	0.16	1.40	0.17	3.76	35.11	7.07
Nodularia spp.	24	0.79	0.23	0.29	1.40	0.17	3.76	35.11	7.07
Oocystis spp.	24	1.08	0.23	0.48	1.40	0.17	3.76	35.11	7.07
Peridiniella catenata	24	0.77	0.29	0.08	1.40	0.17	3.76	35.11	7.07
Plagioselmis prolonga	24	1.04	0.23	0.45	1.40	0.17	3.76	35.11	7.07
Planctonema lauterbornii	23	0.78	0.23	0.43	1.37	0.17	3.84	35.42	6.78
Planktolyngbya spp.	24	1.00	0.22	0.47	1.40	0.17	3.76	35.11	7.07
Protoperidinium bipes	24	0.83	0.29	0.09	1.40	0.17	3.76	35.11	7.07
Protoperidinium brevipes	23	1.03	0.28	0.16	1.37	0.17	3.84	35.42	6.78
Pseudopedinella spp.	24	0.85	0.25	0.17	1.40	0.17	3.76	35.11	7.07
Pyramimonas spp.	24	0.79	0.25	0.23	1.40	0.17	3.76	35.11	7.07
Skeletonema marinoi	24	0.70	0.30	0.08	1.40	0.17	3.76	35.11	7.07
Snowella spp.	24	1.03	0.23	0.45	1.40	0.17	3.76	35.11	7.07
Teleaulax spp.	24	1.07	0.22	0.47	1.40	0.17	3.76	35.11	7.07
Telonema subtile	24	0.75	0.25	0.19	1.40	0.17	3.76	35.11	7.07
Thalassiosira baltica	24	0.70	0.30	0.07	1.40	0.17	3.76	35.11	7.07
Woronichinia spp.	24	0.95	0.22	0.39	1.40	0.17	3.76	35.11	7.07

Table SIII: All candidate models (Model) for the investigated taxa including the parameter values for the environmental covariates (β_2), the partial trend (β_1), the observation error (σ^2_{Proc}), and the density dependence (φ), together with their SE, followed by the fixed parameters (σ^2_{Obs1} and y_1), log likelihood (logLik), number of estimated parameters (k), length of time series (n), convergence performance (C; 0 = convergence, 1 = no convergence), and ΔAICc. All SE in the table have been calculated using the Hessian.

Taxon	Model	β_2	SE	β_1	SE	φ	SE	σ^2_{Proc}	SE	σ^2_{Obs1}	y_1	lnL	k	n	C	ΔAICc
Amphidinium crassum	year	NA	0.20	−0.11	0.20	−0.32	0.25	0.46	0.20	0.51	−0.89	−29.27	3	24	0	0
Amphidinium crassum	strat	−0.29	0.18	−0.16	0.19	−0.23	0.24	0.39	0.17	0.51	−0.89	−28.07	4	24	0	0.50
Amphidinium crassum	temp	−0.22	0.22	0.00	0.22	−0.21	0.27	0.44	0.19	0.51	−0.89	−28.83	4	24	0	2.03
Amphidinium crassum	sal	0.13	0.18	−0.10	0.20	−0.26	0.26	0.45	0.20	0.51	−0.89	−29.03	4	24	0	2.44
Aphanizomenon spp.	temp	0.20	0.10	0.15	0.10	−0.10	0.24	0.11	0.04	0.06	−0.63	−12.59	4	24	0	0
Aphanizomenon spp.	year	NA	0.10	0.23	0.10	−0.05	0.27	0.14	0.05	0.06	−0.63	−14.47	3	24	0	0.86
Aphanizomenon spp.	strat	−0.06	0.09	0.21	0.11	−0.04	0.27	0.14	0.05	0.06	−0.63	−14.28	4	24	0	3.38
Aphanizomenon spp.	sal	−0.02	0.09	0.23	0.10	−0.05	0.27	0.14	0.05	0.06	−0.63	−14.43	4	24	0	3.68
Chaetoceros spp.	DIP	−0.27	0.12	−0.05	0.13	−0.09	0.29	0.15	0.08	0.15	0.28	−22.47	4	24	0	0
Chaetoceros spp.	strat	0.23	0.11	−0.14	0.13	−0.23	0.28	0.16	0.08	0.15	0.28	−22.90	4	24	0	0.86
Chaetoceros spp.	year	NA	0.14	−0.14	0.14	−0.15	0.32	0.24	0.10	0.15	0.28	−24.85	3	24	0	1.84
Chaetoceros spp.	sal	−0.21	0.11	−0.19	0.13	−0.27	0.28	0.16	0.08	0.15	0.28	−23.48	4	24	0	2.01
Chaetoceros spp.	DIN	−0.10	0.13	−0.12	0.14	−0.11	0.32	0.23	0.10	0.15	0.28	−24.57	4	24	0	4.19
Chaetoceros spp.	ice	0.08	0.13	−0.14	0.14	−0.19	0.31	0.23	0.10	0.15	0.28	−24.67	4	24	0	4.40
Chaetoceros spp.	SiO_4	−0.10	0.18	−0.07	0.20	−0.16	0.33	0.22	0.10	0.15	0.28	−24.69	4	24	0	4.42
Chaetoceros spp.	temp	−0.09	0.15	−0.11	0.16	−0.22	0.31	0.23	0.10	0.15	0.28	−24.69	4	24	0	4.43
Chrysochromulina spp.	year	NA	0.18	0.22	0.18	0.02	0.23	0.54	0.18	0.52	−0.67	−28.48	3	24	0	0
Chrysochromulina spp.	sal	0.14	0.17	0.22	0.18	0.06	0.24	0.53	0.17	0.52	−0.67	−28.12	4	24	0	2.20
Chrysochromulina spp.	strat	−0.14	0.16	0.20	0.18	0.02	0.23	0.53	0.17	0.52	−0.67	−28.13	4	24	0	2.21
Chrysochromulina spp.	temp	0.07	0.20	0.20	0.19	0.01	0.24	0.54	0.18	0.52	−0.67	−28.42	4	24	0	2.79
Dinobryon faculiferum	sal	0.49	0.19	−0.15	0.18	0.48	0.18	0.51	0.24	1.02	−1.14	−30.92	4	23	0	0
Dinobryon faculiferum	temp	−0.60	0.24	0.17	0.24	0.53	0.18	0.54	0.25	1.02	−1.14	−31.19	4	23	0	0.54
Dinobryon faculiferum	strat	−0.46	0.22	−0.20	0.20	0.41	0.20	0.62	0.27	1.02	−1.14	−31.94	4	23	0	2.04
Dinobryon faculiferum	year	NA	0.21	−0.18	0.21	0.47	0.22	0.76	0.32	1.02	−1.14	−33.94	3	23	0	3.08
Dinobryon faculiferum	ice	0.31	0.23	−0.14	0.21	0.47	0.21	0.71	0.30	1.02	−1.14	−33.07	4	23	0	4.30
Dinobryon faculiferum	DIN	−0.30	0.24	−0.10	0.22	0.43	0.21	0.72	0.30	1.02	−1.14	−33.16	4	23	0	4.47
Dinobryon faculiferum	DIP	0.05	0.26	−0.20	0.24	0.46	0.23	0.76	0.32	1.02	−1.14	−33.93	4	23	0	6.01
Dinophysis acuminata	temp	0.17	0.10	0.10	0.08	0.08	0.26	0.06	0.03	0.08	0.49	−9.09	4	24	0	0
Dinophysis acuminata	year	NA	0.08	0.14	0.08	0.14	0.32	0.07	0.04	0.08	0.49	−10.56	3	24	0	0.03
Dinophysis acuminata	sal	−0.13	0.08	0.15	0.09	−0.13	0.32	0.07	0.04	0.08	0.49	−9.63	4	24	0	1.08
Dinophysis acuminata	strat	0.09	0.09	0.16	0.09	−0.04	0.33	0.07	0.04	0.08	0.49	−10.11	4	24	0	2.04
Dolichospermum spp.	year	NA	0.20	−0.23	0.20	0.07	0.22	0.71	0.24	0.26	−1.27	−32.05	3	24	0	0
Dolichospermum spp.	sal	0.16	0.20	−0.24	0.20	0.00	0.23	0.69	0.23	0.26	−1.27	−31.73	4	24	0	2.26
Dolichospermum spp.	strat	−0.16	0.20	−0.27	0.20	0.05	0.22	0.70	0.24	0.26	−1.27	−31.73	4	24	0	2.27
Dolichospermum spp.	temp	0.01	0.23	−0.23	0.22	0.07	0.22	0.71	0.24	0.26	−1.27	−32.05	4	24	0	2.90
Ebria tripartita	strat	−0.32	0.14	0.02	0.13	0.56	0.21	0.31	0.12	0.12	0.28	−24.40	4	24	0	0
Ebria tripartita	year	NA	0.14	0.06	0.14	0.51	0.25	0.41	0.15	0.12	0.28	−26.81	3	24	0	1.93
Ebria tripartita	temp	−0.19	0.17	0.13	0.15	0.46	0.24	0.38	0.14	0.12	0.28	−26.15	4	24	0	3.50
Ebria tripartita	SiO_4	0.20	0.21	−0.07	0.20	0.59	0.25	0.39	0.15	0.12	0.28	−26.37	4	24	0	3.95
Ebria tripartita	sal	−0.07	0.16	0.06	0.14	0.47	0.25	0.42	0.15	0.12	0.28	−26.71	4	24	0	4.63
Eutreptiella spp.	temp	0.69	0.10	−0.23	0.10	−0.04	0.13	0.07	0.04	0.13	0.89	−13.45	4	24	0	0
Eutreptiella spp.	sal	−0.49	0.11	0.01	0.12	−0.27	0.19	0.14	0.07	0.13	0.89	−19.43	4	24	0	11.95
Eutreptiella spp.	strat	0.46	0.13	0.14	0.13	−0.21	0.20	0.19	0.08	0.13	0.89	−20.76	4	24	0	14.61
Eutreptiella spp.	year	NA	0.15	0.07	0.15	0.07	0.25	0.36	0.13	0.13	0.89	−25.77	3	24	0	21.73

Hemiselmis spp.	strat	−0.33	0.16	−0.56	0.20	0.60	0.16	0.35	0.14	0.10	0.35	−25.64	4	24	0	0
Hemiselmis spp.	year	NA	0.22	−0.61	0.22	0.53	0.18	0.42	0.16	0.10	0.35	−27.79	3	24	0	1.39
Hemiselmis spp.	temp	−0.16	0.16	−0.52	0.23	0.58	0.18	0.40	0.15	0.10	0.35	−27.26	4	24	0	3.22
Hemiselmis spp.	sal	0.08	0.16	−0.60	0.23	0.54	0.18	0.42	0.16	0.10	0.35	−27.67	4	24	0	4.06
Heterocapsa rotundata	sal	0.29	0.14	−0.18	0.15	−0.15	0.24	0.30	0.11	0.13	0.27	−22.51	4	24	0	0
Heterocapsa rotundata	year	NA	0.15	−0.18	0.15	−0.03	0.26	0.36	0.13	0.13	0.27	−24.59	3	24	0	1.26
Heterocapsa rotundata	strat	−0.24	0.14	−0.22	0.15	−0.17	0.25	0.31	0.12	0.13	0.27	−23.19	4	24	0	1.37
Heterocapsa rotundata	temp	−0.21	0.16	−0.16	0.15	−0.17	0.27	0.33	0.13	0.13	0.27	−23.74	4	24	0	2.47
Heterocapsa triquetra	temp	−0.59	0.30	0.31	0.31	−0.16	0.21	1.57	0.52	0.55	−0.46	−39.49	4	23	0	0
Heterocapsa triquetra	year	NA	0.32	0.13	0.32	−0.17	0.22	1.87	0.61	0.55	−0.46	−41.21	3	23	0	0.49
Heterocapsa triquetra	sal	0.48	0.31	0.17	0.30	−0.06	0.23	1.67	0.55	0.55	−0.46	−40.07	4	23	0	1.17
Heterocapsa triquetra	strat	−0.36	0.31	0.06	0.31	−0.10	0.23	1.75	0.57	0.55	−0.46	−40.55	4	23	0	2.14
Katablepharis spp.	year	NA	0.13	−0.19	0.13	0.19	0.33	0.19	0.11	0.34	−0.85	−22.94	3	24	0	0
Katablepharis spp.	DIP	0.19	0.14	−0.25	0.13	0.23	0.32	0.16	0.10	0.34	−0.85	−21.97	4	24	0	0.96
Katablepharis spp.	sal	−0.15	0.12	−0.19	0.12	0.25	0.31	0.17	0.10	0.34	−0.85	−22.25	4	24	0	1.53
Katablepharis spp.	temp	0.11	0.16	−0.24	0.14	0.27	0.34	0.17	0.11	0.34	−0.85	−22.70	4	24	0	2.43
Katablepharis spp.	DIN	0.08	0.14	−0.20	0.13	0.23	0.35	0.18	0.11	0.34	−0.85	−22.79	4	24	0	2.60
Katablepharis spp.	strat	0.07	0.15	−0.17	0.13	0.27	0.37	0.18	0.11	0.34	−0.85	−22.84	4	24	0	2.70
Katablepharis spp.	ice	−0.04	0.13	−0.19	0.13	0.21	0.33	0.19	0.11	0.34	−0.85	−22.89	4	24	0	2.80
Mesodinium rubrum	sal	−0.22	0.12	0.61	0.26	0.45	0.18	0.23	0.11	0.63	−4.26	−25.68	4	24	0	0
Mesodinium rubrum	year	NA	0.29	0.65	0.29	0.43	0.19	0.27	0.12	0.63	−4.26	−27.14	3	24	0	0.02
Mesodinium rubrum	DIN	−0.18	0.14	0.69	0.28	0.44	0.19	0.26	0.12	0.63	−4.26	−26.36	4	24	0	1.35
Mesodinium rubrum	strat	0.16	0.13	0.63	0.28	0.45	0.19	0.25	0.12	0.63	−4.26	−26.47	4	24	0	1.57
Mesodinium rubrum	temp	0.12	0.16	0.56	0.30	0.45	0.19	0.25	0.12	0.63	−4.26	−26.90	4	24	0	2.43
Mesodinium rubrum	ice	0.10	0.15	0.71	0.30	0.40	0.20	0.27	0.13	0.63	−4.26	−26.92	4	24	0	2.48
Mesodinium rubrum	DIP	−0.07	0.15	0.67	0.29	0.43	0.20	0.27	0.13	0.63	−4.26	−27.03	4	24	0	2.70
Monoraphidium contortum	year	NA	0.17	0.03	0.17	0.42	0.20	0.61	0.21	0.13	−1.36	−30.12	3	24	0	0
Monoraphidium contortum	DIN	−0.29	0.17	0.08	0.16	0.48	0.19	0.51	0.18	0.13	−1.36	−28.78	4	24	0	0.22
Monoraphidium contortum	temp	0.23	0.21	−0.09	0.20	0.36	0.21	0.59	0.20	0.13	−1.36	−29.58	4	24	0	1.82
Monoraphidium contortum	ice	−0.15	0.18	0.01	0.17	0.40	0.20	0.60	0.20	0.13	−1.36	−29.78	4	24	0	2.22
Monoraphidium contortum	DIP	0.14	0.19	−0.02	0.18	0.44	0.20	0.59	0.20	0.13	−1.36	−29.84	4	24	0	2.34
Monoraphidium contortum	sal	−0.14	0.18	0.03	0.17	0.38	0.21	0.60	0.20	0.13	−1.36	−29.85	4	24	0	2.36
Monoraphidium contortum	strat	−0.02	0.18	0.02	0.17	0.43	0.20	0.61	0.21	0.13	−1.36	−30.12	4	24	0	2.89
Nodularia spp.	year	NA	0.26	−0.43	0.26	−0.03	0.24	0.93	0.33	0.31	−0.09	−36.69	3	24	0	0
Nodularia spp.	strat	−0.19	0.22	−0.44	0.25	0.00	0.24	0.91	0.32	0.31	−0.09	−36.35	4	24	0	2.23
Nodularia spp.	sal	0.16	0.22	−0.42	0.26	−0.03	0.24	0.92	0.33	0.31	−0.09	−36.41	4	24	0	2.35
Nodularia spp.	temp	−0.04	0.26	−0.41	0.29	−0.02	0.25	0.94	0.33	0.31	−0.09	−36.67	4	24	0	2.88
Oocystis spp.	year	NA	0.14	−0.04	0.14	0.53	0.19	0.35	0.13	0.07	0.89	−24.62	3	24	0	0
Oocystis spp.	strat	−0.23	0.14	−0.06	0.13	0.52	0.18	0.29	0.11	0.07	0.89	−23.30	4	24	0	0.26
Oocystis spp.	temp	−0.19	0.14	0.01	0.13	0.58	0.18	0.30	0.12	0.07	0.89	−23.69	4	24	0	1.06
Oocystis spp.	sal	−0.10	0.14	−0.06	0.14	0.52	0.19	0.35	0.13	0.07	0.89	−24.39	4	24	0	2.46
Peridiniella catenata	DIN	−0.26	0.15	0.26	0.08	0.92	0.16	0.00	0.02	0.38	1.52	−25.98	4	24	1	0
Peridiniella catenata	year	NA	0.06	0.12	0.06	0.86	0.15	0.00	0.02	0.38	1.52	−27.75	3	24	1	0.64
Peridiniella catenata	ice	−0.21	0.13	0.14	0.06	0.93	0.16	0.00	0.02	0.38	1.52	−26.57	4	24	1	1.19
Peridiniella catenata	temp	0.19	0.14	0.05	0.10	0.91	0.16	0.00	0.02	0.38	1.52	−26.79	4	24	1	1.64
Peridiniella catenata	DIP	0.21	0.19	0.06	0.08	0.87	0.16	0.00	0.02	0.38	1.52	−27.06	4	24	1	2.17
Peridiniella catenata	sal	0.10	0.13	0.14	0.06	0.86	0.16	0.00	0.02	0.38	1.52	−27.42	4	24	1	2.88
Peridiniella catenata	strat	−0.10	0.15	0.13	0.06	0.90	0.16	0.00	0.02	0.38	1.52	−27.51	4	24	1	3.07
Plagioselmis prolonga	year	NA	0.12	0.01	0.12	0.31	0.22	0.27	0.09	0.05	0.29	−20.08	3	24	0	0
Plagioselmis prolonga	strat	−0.17	0.12	0.00	0.11	0.27	0.21	0.25	0.09	0.05	0.29	−19.08	4	24	0	0.90
Plagioselmis prolonga	sal	0.13	0.12	0.02	0.11	0.31	0.21	0.26	0.09	0.05	0.29	−19.49	4	24	0	1.72
Plagioselmis prolonga	temp	0.02	0.12	0.01	0.12	0.31	0.22	0.27	0.09	0.05	0.29	−20.08	4	24	0	2.89
Planctonema lauterbornii	year	NA	0.23	−0.47	0.23	0.55	0.13	0.98	0.38	6.21	−5.76	−37.14	3	23	0	0
Planctonema lauterbornii	temp	0.38	0.26	−0.64	0.25	0.55	0.12	0.86	0.34	6.21	−5.76	−36.14	4	23	0	0.95
Planctonema lauterbornii	strat	0.18	0.28	−0.44	0.23	0.57	0.13	0.94	0.37	6.21	−5.76	−36.95	4	23	0	2.57

Planctonema lauterbornii	sal	−0.12	0.27	−0.47	0.23	0.56	0.13	0.96	0.37	6.21	−5.76	−37.04	4	23	0	2.75
Planktolyngbya spp.	year	NA	0.19	0.06	0.19	0.53	0.18	0.63	0.24	0.44	−2.35	−31.68	3	23	0	0
Planktolyngbya spp.	strat	0.20	0.21	0.10	0.19	0.55	0.18	0.62	0.24	0.44	−2.35	−31.22	4	23	0	2.03
Planktolyngbya spp.	temp	0.19	0.20	0.01	0.19	0.51	0.18	0.63	0.24	0.44	−2.35	−31.25	4	23	0	2.09
Planktolyngbya spp.	sal	−0.18	0.20	0.08	0.19	0.53	0.18	0.63	0.24	0.44	−2.35	−31.28	4	23	0	2.16
Protoperidinium bipes	sal	0.45	0.12	−0.08	0.11	0.46	0.17	0.12	0.09	0.17	1.22	−20.60	4	24	0	0
Protoperidinium bipes	DIP	0.29	0.16	−0.22	0.15	0.49	0.23	0.22	0.13	0.17	1.22	−24.67	4	24	0	8.14
Protoperidinium bipes	year	NA	0.15	−0.10	0.15	0.55	0.24	0.27	0.15	0.17	1.22	−26.21	3	24	0	8.32
Protoperidinium bipes	temp	−0.24	0.16	0.01	0.16	0.55	0.23	0.23	0.14	0.17	1.22	−25.21	4	24	0	9.23
Protoperidinium bipes	strat	−0.16	0.17	−0.13	0.16	0.45	0.26	0.27	0.15	0.17	1.22	−25.80	4	24	0	10.4
Protoperidinium bipes	ice	0.14	0.16	−0.09	0.15	0.56	0.24	0.25	0.15	0.17	1.22	−25.83	4	24	0	10.46
Protoperidinium bipes	DIN	−0.04	0.16	−0.09	0.16	0.55	0.24	0.27	0.15	0.17	1.22	−26.18	4	24	0	11.16
Protoperidinium brevipes	year	NA	0.28	0.63	0.28	0.14	0.31	0.52	0.22	1.33	−0.95	−30.93	3	22	0	0
Protoperidinium brevipes	DIN	−0.29	0.18	0.66	0.26	0.20	0.29	0.43	0.19	1.33	−0.95	−29.79	4	22	0	0.74
Protoperidinium brevipes	ice	−0.16	0.21	0.64	0.28	0.10	0.30	0.52	0.22	1.33	−0.95	−30.62	4	22	0	2.41
Protoperidinium brevipes	DIP	−0.18	0.23	0.65	0.28	0.21	0.30	0.49	0.21	1.33	−0.95	−30.64	4	22	0	2.44
Protoperidinium brevipes	strat	−0.08	0.23	0.58	0.31	0.22	0.37	0.50	0.22	1.33	−0.95	−30.88	4	22	0	2.92
Protoperidinium brevipes	sal	−0.03	0.20	0.64	0.29	0.13	0.34	0.52	0.22	1.33	−0.95	−30.91	4	22	0	2.99
Protoperidinium brevipes	temp	0.00	0.26	0.63	0.29	0.14	0.34	0.52	0.22	1.33	−0.95	−30.93	4	22	0	3.02
Pseudopedinella spp.	sal	−0.32	0.11	0.10	0.10	0.67	0.13	0.18	0.08	0.35	−0.40	−18.26	4	24	0	0
Pseudopedinella spp.	temp	0.30	0.14	−0.07	0.13	0.76	0.14	0.20	0.09	0.35	−0.40	−20.21	4	24	0	3.91
Pseudopedinella spp.	year	NA	0.12	0.10	0.12	0.73	0.15	0.26	0.11	0.35	−0.40	−22.19	3	24	0	4.95
Pseudopedinella spp.	strat	0.17	0.13	0.14	0.12	0.70	0.15	0.25	0.11	0.35	−0.40	−21.32	4	24	0	6.13
Pyramimonas spp.	year	NA	0.13	0.27	0.13	0.00	0.24	0.24	0.08	0.09	−0.79	−19.13	3	24	0	0
Pyramimonas spp.	temp	−0.11	0.13	0.30	0.14	0.03	0.24	0.23	0.08	0.09	−0.79	−18.75	4	24	0	2.15
Pyramimonas spp.	strat	−0.09	0.11	0.26	0.13	−0.01	0.24	0.23	0.08	0.09	−0.79	−18.79	4	24	0	2.24
Pyramimonas spp.	sal	−0.05	0.11	0.27	0.13	0.00	0.24	0.23	0.08	0.09	−0.79	−19.03	4	24	0	2.71
Skeletonema marinoi	year	NA	0.20	−0.25	0.20	−0.22	0.27	0.58	0.21	0.18	0.76	−30.40	3	24	0	0
Skeletonema marinoi	DIN	0.24	0.18	−0.29	0.20	−0.29	0.26	0.55	0.21	0.18	0.76	−29.60	4	24	0	1.29
Skeletonema marinoi	ice	−0.20	0.18	−0.25	0.19	−0.22	0.25	0.54	0.20	0.18	0.76	−29.80	4	24	0	1.69
Skeletonema marinoi	DIP	−0.13	0.19	−0.21	0.20	−0.21	0.26	0.56	0.21	0.18	0.76	−30.17	4	24	0	2.43
Skeletonema marinoi	SiO_4	0.10	0.25	−0.32	0.26	−0.21	0.27	0.58	0.21	0.18	0.76	−30.32	4	24	0	2.73
Skeletonema marinoi	sal	−0.04	0.18	−0.26	0.20	−0.22	0.27	0.58	0.21	0.18	0.76	−30.38	4	24	0	2.86
Skeletonema marinoi	temp	−0.04	0.21	−0.23	0.22	−0.23	0.27	0.58	0.21	0.18	0.76	−30.39	4	24	0	2.87
Skeletonema marinoi	strat	−0.03	0.18	−0.25	0.20	−0.22	0.27	0.58	0.21	0.18	0.76	−30.39	4	24	0	2.88
Snowella spp.	strat	−0.58	0.24	−1.06	0.40	0.37	0.21	0.70	0.32	0.26	1.94	−35.22	4	24	0	0
Snowella spp.	temp	−0.54	0.23	−0.84	0.38	0.38	0.21	0.62	0.29	0.26	1.94	−35.23	4	24	0	0.02
Snowella spp.	year	NA	0.43	−0.84	0.43	0.47	0.23	0.84	0.37	0.26	1.94	−37.97	3	24	0	2.59
Snowella spp.	sal	0.39	0.23	−0.84	0.41	0.46	0.22	0.72	0.33	0.26	1.94	−36.52	4	24	0	2.60
Teleaulax spp.	sal	0.19	0.10	−0.03	0.11	0.44	0.22	0.19	0.07	0.04	0.50	−15.96	4	24	0	0
Teleaulax spp.	strat	−0.19	0.10	−0.07	0.11	0.39	0.22	0.19	0.07	0.04	0.50	−16.09	4	24	0	0.25
Teleaulax spp.	year	NA	0.12	−0.04	0.12	0.47	0.23	0.22	0.08	0.04	0.50	−17.64	3	24	0	0.44
Teleaulax spp.	temp	−0.01	0.11	−0.04	0.12	0.47	0.24	0.22	0.08	0.04	0.50	−17.64	4	24	0	3.35
Telonema subtile	year	NA	0.14	−0.04	0.14	0.35	0.33	0.28	0.16	0.26	0.50	−26.30	3	24	0	0
Telonema subtile	temp	−0.26	0.18	0.04	0.16	0.19	0.33	0.26	0.15	0.26	0.50	−25.35	4	24	0	1.01
Telonema subtile	sal	−0.17	0.14	−0.03	0.13	0.42	0.31	0.23	0.14	0.26	0.50	−25.62	4	24	0	1.55
Telonema subtile	ice	0.03	0.16	−0.04	0.14	0.34	0.33	0.29	0.16	0.26	0.50	−26.29	4	24	0	2.87
Telonema subtile	DIN	−0.02	0.17	−0.04	0.15	0.33	0.34	0.28	0.16	0.26	0.50	−26.30	4	24	0	2.90
Telonema subtile	strat	−0.01	0.16	−0.05	0.15	0.34	0.33	0.28	0.16	0.26	0.50	−26.30	4	24	0	2.90
Telonema subtile	DIP	0.00	0.17	−0.04	0.15	0.35	0.33	0.28	0.16	0.26	0.50	−26.30	4	24	0	2.90
Thalassiosira baltica	ice	−0.54	0.14	−0.28	0.14	−0.14	0.19	0.13	0.10	0.16	1.24	−22.03	4	24	0	0
Thalassiosira baltica	DIP	0.40	0.16	−0.31	0.16	0.10	0.26	0.21	0.12	0.16	1.24	−25.38	4	24	0	6.70
Thalassiosira baltica	SiO_4	0.46	0.19	−0.56	0.21	0.00	0.27	0.22	0.12	0.16	1.24	−25.62	4	24	0	7.19
Thalassiosira baltica	temp	0.41	0.17	−0.47	0.19	−0.10	0.26	0.25	0.13	0.16	1.24	−25.65	4	24	0	7.25
Thalassiosira baltica	strat	−0.34	0.15	−0.20	0.16	0.02	0.27	0.24	0.13	0.16	1.24	−25.82	4	24	0	7.57

Thalassiosira baltica	year	NA	0.19	−0.26	0.19	−0.10	0.31	0.35	0.16	0.16	1.24	−28.29	3	24	0	9.61
Thalassiosira baltica	DIN	0.22	0.16	−0.31	0.19	−0.24	0.28	0.31	0.15	0.16	1.24	−27.40	4	24	0	10.74
Thalassiosira baltica	sal	−0.02	0.17	−0.27	0.21	−0.13	0.33	0.35	0.16	0.16	1.24	−28.28	4	24	0	12.50
Woronichinia spp.	strat	−0.63	0.30	−0.59	0.33	0.38	0.22	1.24	0.48	0.18	2.42	−40.04	4	24	0	0
Woronichinia spp.	temp	0.68	0.32	−1.02	0.40	0.27	0.22	1.20	0.46	0.18	2.42	−40.08	4	24	0	0.08
Woronichinia spp.	year	NA	0.37	−0.58	0.37	0.28	0.24	1.51	0.55	0.18	2.42	−42.13	3	24	0	1.26
Woronichinia spp.	sal	0.35	0.31	−0.60	0.36	0.31	0.24	1.45	0.54	0.18	2.42	−41.51	4	24	0	2.94

Table SIV: Parameter estimates, standard errors and p−values (α = 0.05) from the observation model for the wind effect (Wind), method (FB) and the interaction term (W:FB), as well as the Tweedie power parameter value (Twd). The table also includes the mean log-wind level ($\bar{x}$) and its SD.

Taxon	Wind	se	p	W:FB	se	p	FB	se	p	Twd	$\bar{x}$	SD
Amphidinium crassum	−0.061	0.109	0.577	−0.002	0.142	0.989	−0.710	0.237	0.003	1.460	5.38	2.08
Aphanizomenon spp.	0.005	0.046	0.917	0.096	0.058	0.097	−0.211	0.126	0.095	1.605	5.38	2.08
Chaetoceros spp.	0.182	0.080	0.024	−0.056	0.119	0.636	−0.447	0.208	0.032	1.633	5.38	2.08
Chrysochromulina spp.	0.010	0.063	0.869	−0.266	0.079	0.001	0.062	0.096	0.519	1.671	5.40	2.10
Dinobryon faculiferum	0.137	0.114	0.229	−0.184	0.163	0.259	−0.937	0.328	0.004	1.515	5.38	2.08
Dinophysis acuminata	−0.033	0.066	0.615	0.109	0.082	0.182	0.222	0.146	0.128	1.332	5.38	2.08
Dolichospermum spp.	−0.052	0.084	0.534	0.080	0.098	0.414	−0.011	0.172	0.950	1.583	5.38	2.08
Ebria tripartita	−0.239	0.081	0.003	0.551	0.098	0.000	0.376	0.131	0.004	1.437	5.38	2.09
Eutreptiella spp.	0.086	0.073	0.244	0.211	0.093	0.024	0.311	0.189	0.101	1.653	5.38	2.08
Hemiselmis spp.	−0.067	0.053	0.209	0.181	0.067	0.007	−0.378	0.266	0.156	1.505	5.38	2.08
Heterocapsa rotundata	0.010	0.067	0.884	−0.163	0.089	0.066	−0.182	0.238	0.443	1.624	5.38	2.08
Heterocapsa triquetra	0.102	0.095	0.284	−0.154	0.115	0.182	0.464	0.315	0.141	1.636	5.38	2.08
Katablepharis spp.	0.144	0.074	0.052	−0.061	0.110	0.582	−0.362	0.410	0.377	1.439	5.38	2.08
Mesodinium rubrum	−0.002	0.059	0.971	−0.031	0.089	0.728	−0.435	0.373	0.243	1.556	5.38	2.08
Monoraphidium contortum	0.009	0.051	0.853	0.115	0.070	0.102	0.702	0.302	0.020	1.672	5.37	2.08
Nodularia spp.	0.217	0.103	0.035	−0.433	0.129	0.001	−0.273	0.170	0.109	1.545	5.38	2.08
Oocystis spp.	−0.154	0.066	0.020	0.124	0.084	0.142	0.161	0.146	0.271	1.434	5.38	2.08
Peridiniella catenata	−0.041	0.110	0.713	0.048	0.251	0.849	−0.650	0.448	0.147	1.467	5.38	2.08
Plagioselmis prolonga	0.000	0.043	0.992	−0.014	0.057	0.802	−0.192	0.142	0.176	1.542	5.38	2.08
Planctonema lauterbornii	0.014	0.105	0.891	−0.102	0.134	0.445	0.482	0.275	0.080	1.564	5.38	2.08
Planktolyngbya spp.	−0.001	0.075	0.984	0.208	0.096	0.031	0.363	0.296	0.221	1.504	5.39	2.08
Protoperidinium bipes	0.214	0.084	0.011	−0.213	0.158	0.177	−0.235	0.192	0.220	1.402	5.38	2.08
Protoperidinium brevipes	0.205	0.133	0.122	−0.378	0.177	0.033	0.037	0.205	0.856	1.248	5.40	2.09
Pseudopedinella spp.	0.010	0.050	0.848	−0.146	0.071	0.040	−0.644	0.245	0.009	1.545	5.35	2.00
Pyramimonas spp.	0.122	0.049	0.013	−0.267	0.065	0.000	−0.069	0.167	0.680	1.639	5.38	2.08
Skeletonema marinoi	0.112	0.092	0.224	−0.210	0.137	0.124	0.320	0.186	0.086	1.660	5.38	2.08
Snowella spp.	0.263	0.090	0.004	−0.159	0.112	0.157	−0.855	0.549	0.120	1.538	5.38	2.08
Teleaulax spp.	−0.055	0.044	0.213	0.150	0.058	0.010	−0.195	0.121	0.107	1.502	5.38	2.08
Telonema subtile	0.196	0.100	0.051	−0.083	0.146	0.572	−1.102	0.394	0.005	1.471	5.38	2.08
Thalassiosira baltica	0.065	0.088	0.457	−0.129	0.172	0.454	−0.027	0.331	0.935	1.436	5.38	2.08
Woronichinia spp.	0.048	0.104	0.641	−0.049	0.138	0.723	−0.596	0.407	0.143	1.574	5.38	2.08
